# In Silico Drug Repositioning to Target the SARS-CoV-2 Main Protease as Covalent Inhibitors Employing a Combined Structure-Based Virtual Screening Strategy of Pharmacophore Models and Covalent Docking

**DOI:** 10.3390/ijms23073987

**Published:** 2022-04-03

**Authors:** Luis Heriberto Vázquez-Mendoza, Humberto L. Mendoza-Figueroa, Juan Benjamín García-Vázquez, José Correa-Basurto, Jazmín García-Machorro

**Affiliations:** 1 Laboratorio de Diseño y Desarrollo de Nuevos Fármacos e Innovación Biotecnológica, Posgrado en Farmacología de la Escuela Superior de Medicina del Instituto Politécnico Nacional, Plan de San Luis y Salvador Díaz Mirón s/n, Casco de Santo Tomás, Ciudad de Mexico 11340, Mexico; luis_94hvm@hotmail.com (L.H.V.-M.); corrjose@gmail.com (J.C.-B.); 2 Cátedras CONACyT-Sección de Estudios de Posgrado e Investigación, Escuela Superior de Medicina, Instituto Politécnico Nacional, Plan de San Luis y Díaz Mirón s/n, Casco de Santo Tomás, Ciudad de Mexico 11340, Mexico; 3 Laboratorio de Medicina de la Conservación, Escuela Superior de Medicina del Instituto Politécnico Nacional, Plan de San Luis y Salvador Díaz Mirón s/n, Casco de Santo Tomás, Ciudad de Mexico 11340, Mexico; jazzgama@hotmail.com

**Keywords:** covalent inhibitors, SARS-CoV-2 M^pro^, pharmacophore modeling, structure-based virtual screening, drug repositioning

## Abstract

The epidemic caused by the SARS-CoV-2 coronavirus, which has spread rapidly throughout the world, requires urgent and effective treatments considering that the appearance of viral variants limits the efficacy of vaccines. The main protease of SARS-CoV-2 (M^pro^) is a highly conserved cysteine proteinase, fundamental for the replication of the coronavirus and with a specific cleavage mechanism that positions it as an attractive therapeutic target for the proposal of irreversible inhibitors. A structure-based strategy combining 3D pharmacophoric modeling, virtual screening, and covalent docking was employed to identify the interactions required for molecular recognition, as well as the spatial orientation of the electrophilic warhead, of various drugs, to achieve a covalent interaction with Cys145 of M^pro^. The virtual screening on the structure-based pharmacophoric map of the SARS-CoV-2 M^pro^ in complex with an inhibitor N3 (reference compound) provided high efficiency by identifying 53 drugs (FDA and DrugBank databases) with probabilities of covalent binding, including N3 (Michael acceptor) and others with a variety of electrophilic warheads. Adding the energy contributions of affinity for non-covalent and covalent docking, 16 promising drugs were obtained. Our findings suggest that the FDA-approved drugs Vaborbactam, Cimetidine, Ixazomib, Scopolamine, and Bicalutamide, as well as the other investigational peptide-like drugs (DB04234, DB03456, DB07224, DB7252, and CMX-2043) are potential covalent inhibitors of SARS-CoV-2 M^pro^.

## 1. Introduction

**Figure 1:**
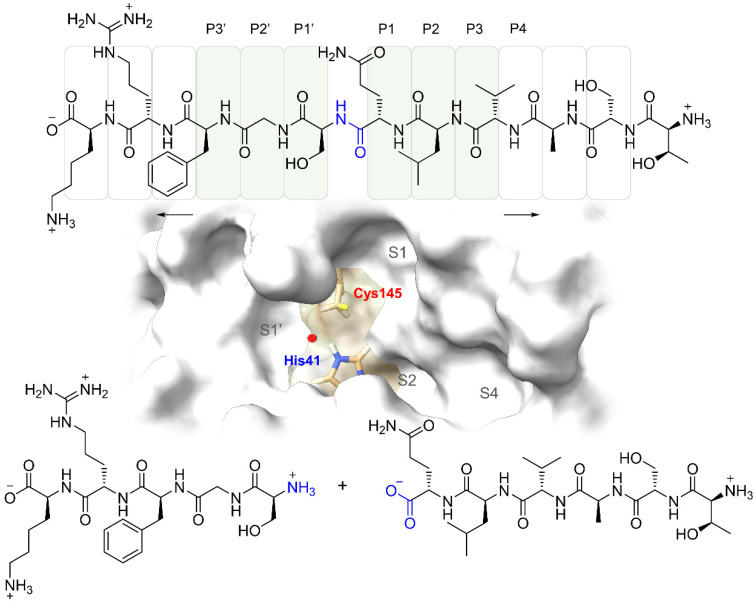
The reaction catalyzed by SARS-CoV-2 M^pro^. Substrate framework regions that are complementary to the substrate binding sites called S1, S1′, S2, and S4; molecular recognition regions in the protease are highlighted. The catalytic dyad constituted by His41 can also be observed; it plays a relevant role in the ionization of the residues favoring the nucleophilic attack of Cys145, on the amide group of the substrate marked in blue, thus completing the hydrolysis of the peptide bond [7].

The recent pandemic outbreak of coronavirus disease 2019 (COVID-19) caused by the new severe acute respiratory syndrome coronavirus 2 (SARS-CoV-2) infected more than 278 million, from which more than 5.4 million died worldwide (as of 28 December 2021) [1]. Coronaviruses are enveloped RNA viruses that are distributed broadly among humans, other mammals, and birds and cause respiratory, enteric, hepatic, and neurologic diseases [2]. Previously, other betacoronavirus-related outbreaks have emerged, including severe acute respiratory syndrome (SARS-CoV) in 2003 and the Middle East respiratory syndrome (MERS-CoV) in 2012, and the molecular knowledge gained about this type of coronavirus was relevant to cope with the global emergency [3]. The structural organization and gene expression of all coronaviruses are similar, involving 16 non-structural proteins (nsp1 to nsp16) and structural proteins such as the nucleocapsid (N), the spike (S), the envelope (E) and the membrane (M). The envelope includes three proteins: the M protein, which binds to the nucleocapsid and enhances virus assembly and budding; protein E, which participates in viral assembly, release and pathogenesis, and protein S, which contributes to homotrimeric peaks that recognize the cellular receptor, favoring the virus invasion of human target cells [4]. Considering the viral structure, several of the structural proteins are positioned as pharmacological targets, and their structural characterization has promoted the design of potent and selective drugs against SARS-CoV-2. Additionally, non-structural proteins participate in the processes of replication and evasion of the immune response, for which reason they are also considered a therapeutic target along with the main protease (M^pro^) of SARS-CoV-2. The M^pro^ protease has functions such as cleaving the polyprotein at 11 different sites, generating many of the non-structural proteins (nsp1 to nsp16), including RNA-dependent RNA polymerase (RdRp) and helicase (Hel), which are important in viral transcription and replication within host cells, being essential in the replication cycle. The structure of M^pro^ has been resolved by crystallography and is that of a homodimeric nucleophilic protease; each protomer consists of three domains and has a catalytic dyad consisting of Cys145 and His41 [5]. The mentioned structural features are shared in 96% sequence identity with SARS-CoV M^pro^, maintaining highly conserved regions that are not mutating despite the environmental adaptation of the virus [6]. Therefore, disrupting the enzyme activity of M^pro^ can stop the processing the virus genome for further assembly in the replication cycle of SARS-CoV-2. SARS-CoV-2 M^pro^ has a unique recognition sequence that is characterized by cleavage of peptides including sequences such as [Asn/Ser/Ala/Gly: P1′] ↓ [P1: Gln] [P2: Leu/Phe/Val/Met] [P3: X] [P4: small], where “small” denotes a non-bulky residue (e.g., Ala, Val, Pro, or Thr), “X” indicates any amino acid, and “↓” indicates the cleavage amide group. Furthermore, it has been established that there is a remarkably high degree of conservation of substrate binding sites, particularly for the crucial S1/S2 subsites [7] (Figure 1).

According to the catalytic process above, no similar human protease is known to have this cleavage specificity; this makes M^pro^ an excellent target for designing drugs with less toxic effects. The M^pro^ inhibitor proposal is in constant development; many of the designed molecules are covalent inhibitors that have an advantage from a pharmacokinetic and pharmacodynamic point of view. Historically, the proposal for covalent inhibitors has been held back due to the fear of non-specific protein inhibition off-target, generating toxicity risks. However, the precise structural knowledge of the enzyme catalytic site and the reactivity predictions of various electrophilic warheads have made it possible to obtain highly selective compounds [8]. Today, covalent inhibitors can be found with a wide variety of electrophilic centers and are used in different therapies. For example, proteasome inhibitors such as epoxomycin are highly active against cancer [9], epoxyketone-derived selective immunoproteasome inhibitors are a promising approach for the treatment of autoimmune disorders [10], while propargylamine and carbamate derivative inhibitors targeting monoamine oxidase A, monoamine oxidase B, and acetylcholinesterase are useful in the central nervous system [11] and cardiovascular disorders [12]. The alpha-fluoromethyl ketone analogs function as covalent inhibitors of human intestinal bacterial bile salt hydrolases [13,14] and, finally, the covalent inhibitors with antiviral activity are those with the greatest structural variability in the electrophilic centers [15]. However, the most abundant indication is in oncology, since 10 of the 14 covalent drugs approved by the FDA between 2011 and 2019 were anticancer drugs [16]. Of note, there has been great interest in the characterization of alternative warheads to achieve selectivity and potency over SARS-CoV-2 M^pro^, although Michael acceptors, alfa-ketoamides, aldehydes and hydroxymethylketones are the predominant warheads in the field of the current development of covalent drugs [17]. The most effective M^pro^ inhibitors identified so far, including the clinical candidates PF-00835231 and PF-07321332, incorporate a glutamine residue or a bioisostere at the P1 position for potency and selectivity supported on a peptidomimetic scaffold endowed with hydrophobic branched substituents in positions P2 and P3 [18]. However, the only M^pro^ inhibitor that has shown efficacy in advanced clinical trials is PF-07321332 [19].

Unlike that of non-covalent inhibitors, the process of molecular recognition of a covalent inhibitor depends not only on the structural complementarity between the catalytic site and the inhibitor, but also on the appropriate chemical reactivity of the electrophilic center and the protein environment that stabilizes the covalent complex. Therefore, designing covalent inhibitors requires understanding the energy contributions of the different steps in the formation of the covalent complex, including both the free energy of non-covalent bonding and the free energies of reaction. In this sense, with the application of computational tools based on quantum mechanics/molecular mechanics (QM/MM), including molecular docking, molecular dynamics (MD), linear-scaling DFT, and others, researchers have been able to establish the structural requirements of the ligands in the non-covalent bonding process (molecular recognition) and subsequently the irreversible bond, which are necessary to slow down the catalytic cycle [7,20,21]. The implementation of these chemoinformatics tools has favored the proposal of drug repurposing, as well as the structural chemical knowledge that promotes drug design.

Drug repurposing, also known as drug repositioning, is a strategy employed in drug development that identifies new medical uses for drugs approved for clinical or investigational use. This has enormous advantages compared to traditional development, as this strategy shortens time-consuming stages in drug development such as safety evaluation and testing, as well as optimization of molecular hits, substantially reducing the investment required [22]. For this reason, drug repositioning became a strategy to deal with the COVID-19 pandemic, finding few drugs that demonstrated their ability to stop viral replication [23,24,25,26,27].

**Figure 2:**
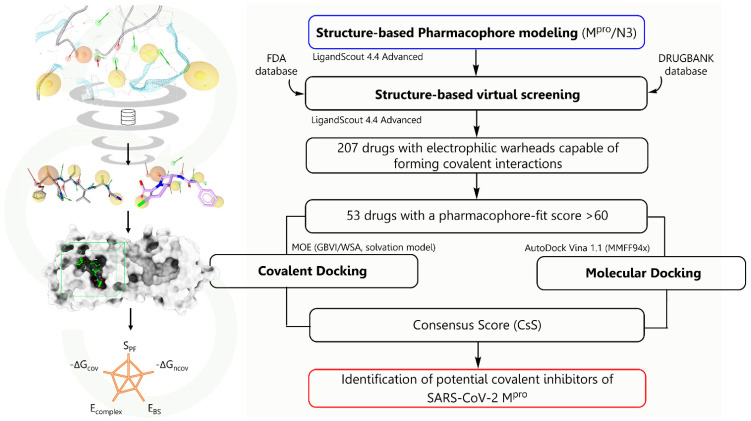
Overview and workflow implemented in the combined structure-based approach with pharmacophoric modeling, virtual screening, non-covalent docking and covalent docking.

Furthermore, in this work we present a bio-targeted guide under a computer-driven approach to the selection of drugs with covalently bound warheads as potential SARS-CoV-2 M^pro^ inhibitors, employing a structure-based strategy that includes high-efficiency virtual screening on 3D pharmacophore models capable of identifying the minimal interactions for non-covalent molecular recognition, as well as the necessary spatial orientation of the electrophilic center for subsequent covalent binding, unlike traditional pharmacophore models (Figure 2). The binding free energies were calculated by molecular traditional docking and finally by covalent docking studies. The databases used were DrugBank and FDA; our approach allowed the identification of drugs approved by the FDA and others under investigation for non-peptidic and peptidomimetic characteristics, respectively. From the best-ranked drugs, we identified five different covalent warheads: boronic acids (Vaborbactam and Ixazomib), carbonitriles (Cimetidine, Bicalutamide and DB03456), epoxides (Scopolamide, DB07224, and DB07225), aldehydes (DB04234), and disulfide bond (CMX-2043). These drugs are positioned as potential inhibitors of SARS-CoV-2 M^pro^.

## 2. Results and Discussion

Under the concept inherent in the design and repositioning of covalent drugs, our search for molecules that could inhibit irreversibly the SARS-CoV-2 M^pro^ was guided with consideration for the two-step mechanism associated with the inhibition of the pharmacological target. Initially, undoubtedly, the ligand will have to bind non-covalently to the catalytic site of the enzyme, promoted by the physicochemical complementarity of functional groups and amino acid residues. This, in turn, guarantees the binding mode that favors the approximation of the reactive centers of the ligand, commonly electrophilic, and of the pharmacological target with its nucleophilic residues (either the thiol group of cysteine or the hydroxyl group of serine). In this second step, the covalent bond is formed in situ, generating a protein–ligand covalent complex [28]. By combining structure-based modeling strategies, we prioritized the order according to the above-mentioned mechanism. Thus, the pharmacophoric modeling of the N3/M^pro^ complex gave us the nature, three-dimensional arrangement, and directionality (hydrogen bonds) of the molecular interactions that determine molecular recognition. Next, we carried out the screening and corroborated the binding affinity of the drugs to the catalytic site by non-covalent docking. Finally, all of those drugs with the best scores were subjected to flexible docking to identify the formation of the covalent complex.

### 2.1. Structure-Based Pharmacophoric Modeling

The pharmacophoric model was generated from the crystal structure of SARS-CoV-2 M^pro^ in complex with an inhibitor called N3. N3 is an irreversible peptidomimetic inhibitor that consists of a Michael-type acceptor electrophilic center and can react with the Cys145 side chain forming a covalent bond that stabilizes the complex. According to its structural characteristics, N3 exhibits a very strong inhibition of SARS-CoV-2 M^pro^ (antiviral activity assay, EC_50_ = 16.77 μM) [29]. The 3D pharmacophore model comprises the essential non-covalent interactions and reflects the structural conformation of N3 required from the rest of the warhead for interaction with the catalytic Cys145 of SARS-CoV-2 M^pro^.

**Figure 3:**
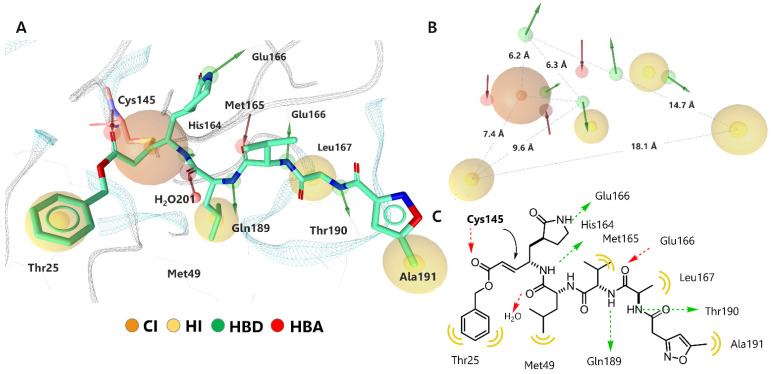
Structure-based pharmacophoric modeling of SARS-CoV-2 M^pro^ in complex with N3. (**A**) Representation of the pharmacophoric modeling on the binding site pocket. (**B**) A 13-point pharmacophoric map; key features include HBD (green), HBA (red), H (yellow), CI (orange), and distance relation in Å. (**C**) Two-dimensional representation of the interactions that determine the binding of the inhibitor N3 on SARS-CoV-2 M^pro^.

The results regarding the structure-based pharmacophoric map are shown in Figure 3; a model is observed with 13 pharmacophoric features in which hydrogen-bonding interactions predominate. Specifically, five hydrogen bond donor interactions (HBD) with amino acids His164, Glu166, Gln189, and Thr190 stand out. Three interactions as hydrogen bond acceptor (HBA) were located with residues Cys145, Glu166 and a structural water molecule HOH201, which is considered crucial in the catalytic cycle of M^pro^ [30]. Four hydrophobic regions (H) were surrounded by Thr25, Met49, Met165, Leu167, and Ala191. These types of molecular interactions observed in the pharmacophoric map of the inhibitor-M^pro^ complex are consistent with other inhibitors of the α-ketoamide type in crystalline complexes, both in the site and mode of binding (in domains I and II of the protease) [31]. Finally, the covalent bond (CI) that is generated between the -SH group of Cys145 and the α, β-unsaturated system of N3, which favors 1,4-addition nucleophilic (Figure 3C), was clearly appreciated.

The binding site of M^pro^ consists of a conserved catalytic dyad that is represented by His41 and Cys145, playing an important role in other residues such as Phe140, Leu141 Asn142, Gly143, Ser144, Cys145, Met165, Glu166, Gln189 and Thr190, which promote complementary interactions favoring the molecular recognition of ligands. Previously, these types of interactions had been identified by structure-based methods including X-ray diffraction [32], molecular dynamics simulations [33], and pharmacophoric modeling [34,35,36,37,38], suggesting a high reliability of the design of our pharmacophoric map focused on the search for covalent inhibitors.

### 2.2. Virtual Screening on the Structure-Based Pharmacophoric Map

In recent years, pharmacophore models and molecular docking techniques have been widely used in virtual screening [39]. Detection of specific and potent inhibitors using pharmacophoric maps is more accurate and rapid due to the alignment of common structural and molecular recognition features at the active site of the therapeutic target [40]. To achieve the identification of covalent fragments, LigandScout 4.4 was used, taking advantage of the fact that this software incorporates the identification of electrophilic centers capable of forming covalent bonds with the nucleophilic residues of the macromolecules. This tool has been successfully applied in the search for covalent inhibitors for different viral proteases [41,42,43].

The generated 3D pharmacophore model was subsequently used to screen the collection of drugs deposited in the FDA and DrugBank databases (approved, investigational, and experimental). The results obtained from the high-throughput virtual screening (see parameters in the Section 3.3) are shown in Table 1, where the drugs are prioritized by the value of fit to the pharmacophoric map. With this first filter, only 53 drugs with a pharmacophore-fit score >60 were selected. It is important to highlight that the pharmacophoric map was able to identify 207 additional drugs with structural fragments capable of forming covalent interactions. However, they were not considered because they presented a low adjustment to the map, lacking the non-covalent interactions relevant for M^pro^ inhibition. Interestingly, the best pharmacophore fit score was achieved with the N3 inhibitor, demonstrating that the initial pharmacophore hypothesis was able to detect all of the required interactions despite the high conformational flexibility of N3. The specificity of the pharmacophoric model was also reflected with the adjustment of drugs N1, N9, I2, Boceprevir, and Telaprevir, since crystallographic data on the covalent complex with SARS-CoV-2 M^pro^ were identified (PDB IDs of crystal complexes N3: 7BQY and 6LU7; N1: 1WDF.; N9: 2AMD.; I2: 2D2D.; Boceprevir: 7NBR, 6XQU, 7C6S, 7BPR, 6WNP, 6ZRU.; Telaprevir: 6XQS, 6ZRT, 7K6D, 7LB7, 7C7P and 7NBS.). Previous data validated our model and demonstrated that the druggability of M^pro^ is possible due to the complementarity of pharmacophoric features with like-peptide and non-peptide drugs in addition to the structural diversity of the electrophilic center of each molecule. Table 1 shows the pharmacophoric characteristics related to the map structure based of M^pro^/N3, as well as the relevant amino acids in molecular recognition. Cys145, Met165, Glu166 and Gln189 were relevant in the interactions exhibited by the best scored drugs. These results are consistent with those obtained in interaction analysis by molecular dynamics simulation of peptide-like inhibitors in SARS-CoV-2 M^pro^ [38]. In the molecular dynamics simulations, the HBD and HBA interaction of the inhibitors with Glu166 maintained the higher rate of interaction, while the interaction of HBD with His41 was maintained over 80% in all of the analyzed ligands. Furthermore, ligands such as Indinavir and other ligands co-crystallized with SARS-CoV M^pro^ interacted with Gly143 and Cys145, with a probability greater than 50% during the simulation. Additionally, we identified the importance of the van de Waals-type interactions generated by the residues Thr25, Met49, Met165, Leu167 and Ala191, as well as the interaction HBA with the water molecule HOH201, since they are involved in the structural stability of the inhibitor/M^pro^ complex [44], as observed in the non-covalent molecular docking results for each drug obtained in the screening. Therefore, according to our results, it is possible to propose the repositioning of peptide-like and non-peptide drugs that adjust with key interactions for the inhibition of SARS-CoV-2 M^pro^, delimited in a designed pharmacophoric map.

In general, we classified the identified drugs into four main groups: (i) antivirals such as N3, N1, N9, Amprenavir, Boceprevir, Telaprevir, Darunavir, Fosamprenavir, Atazanavir, and BMS-488043; (ii) antibiotics such as Ceftaroline, Temocillin, Aztreonam, Cefaloglycin, Mupirocin, and Cefditoren; (iii) proteasome-inhibiting anti-cancer drugs such as Ixazomib, Oprozomib, Carfilzomib, as well as Prexasertib, an inhibitor of checkpoint kinase 1 (chk1); (iv) inhibitors of proteolytic enzymes, Flovagatran, Calpain inhibitor, DB08119, DB03984, DB07224, DB03456, DB07299, etc. (for details, consult Table S2). Specifically, several antiviral drugs have been shown to inhibit M^pro^. For example, Boceprevir shows a strong inhibition of protease (IC_50_ = 1.42 μM) and antiviral activity (EC_50_ = 49.89 μM), while Telaprevir and Nelfinavir show a moderate inhibition of protease (IC_50_ = 11.47 μM) and high antiviral activity (EC_50_ = 3.28 µM), respectively [45,46].

Considering that the 53 identified drugs have the possibility of forming a covalent bound with Cys145, the covalent docking studies were carried out to determine the affinity energy and obtain a final consensus scoring [47].

### 2.3. Validation and Covalent Docking Dependent Virtual Screening

**Figure 4:**
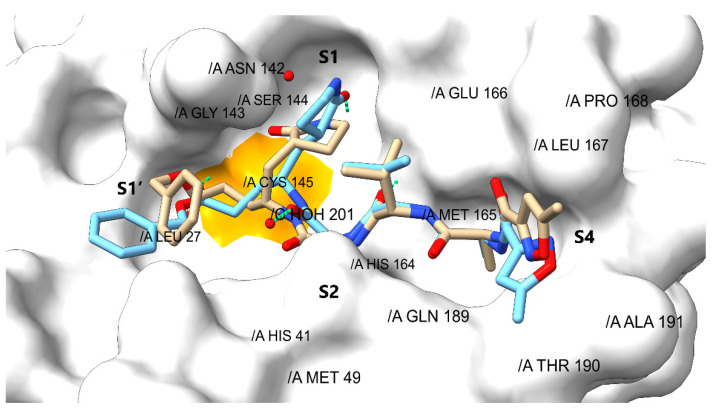
N3 redocking over SARS-CoV-2 M^pro^ (PDB ID: 7BQY). The co-crystallized inhibitor is shown in blue, the MOE-docked inhibitor in brown (RMSD = 0.65), and orange highlights the covalent interaction with Cys145.

Once those drugs that structurally contain a covalent warhead linked to the pharmacophoric hypothesis had been identified, the ability of M^pro^ to couple to the different ligands by flexible docking was evaluated, looking for the formation of the covalent bond according to the catalytic properties of M^pro^. For this, the protocol of the bioinformatics method was validated through the independent docking of the co-crystallized ligand (N3) on the active site, to achieve the bioactive conformation described in the X-ray protein-ligand complex. Consequently, the binding pose of N3 on the M^pro^ catalytic site was the one that showed the lowest energy score, and its binding mode adopted a conformation close to the co-crystallized structure with an RMSD = 0.65 (Figure 4). The molecular recognition of N3 was located in the substrate-binding pocket of M^pro^, which is known to be highly conserved and has been structurally characterized with precision. It is composed of a cleft between domains I and II with sub-pockets named as S1, S1′, S2 and S4 (Figure S1) [48]. As expected, the relevant interactions were observed between Cys145 side chain and N3, as well as with His41, alkyl and π-σ interactions with the pyrrolidinone substituent of N3. Complementarity with these two amino acid residues is crucial to achieving the M^pro^ inhibitory effect, since they are relevant in the catalytic mechanism of M^pro^ and responsible for the stabilization of the covalent complex [49].

Likewise, interactions were observed by hydrogen bonding with amino acids located in the center of the sub-pockets specifically between His164, Glu166, and Gln189 and the L-alanyl-L-valyl region of N3, also with Asn 142, Gly143, and Ser144 (located in S1′ and S2), and finally with Thr190 of S1. Adjacently, hydrophobic interactions were observed in the S3 and S4 sub-pocket with the Met165, Leu167, Pro168, and Ala191 residues (Figure 4 and Figure S1). A surprising degree of similarity was observed between the pharmacophoric entities found in the 3D map; the molecular interactions analyzed by covalent docking and in the crystalline complexes revealed the high resolution that the virtual screening based on pharmacophoric maps could provide. These data suggest a considerable increase in the anchoring precision of new drugs despite the dynamic properties of the M^pro^ sub-pockets; this fact could be verified in a recent study [34]. Consequently, in addition to the parameters calculated in the virtual screening, we added the binding energy and the interactions obtained by covalent docking, reaching a final energy value calculated by the consensus score equation. Thus, Table S3 shows the results of all drugs subjected to covalent docking, while in Table 2 only those drugs with the best scores were recovered, without considering those that have previously shown inhibitory activity experimentally.

Therefore, 16 drugs were selected, considering as the cut-off point the last best positioned antiviral with experimental inhibition data, which in this case corresponded to Amprenavir (the chemical structures of all the drugs can be consulted in Table S4). Specifically, the final list presented seven drugs approved by the FDA with structural and pharmacological diversity, corresponding to Vaborbactam, Cimetidine, Ixazomib, Scopolamine, Bicalutamide, Prexasertib and Riociguat. For example, Vaborbactam and Ixazomib have a boron atom capable of forming the covalent interaction, and Cimetidine and Bicalutamide are sensitive to nucleophilic attack on the carbonitrile substituent, while Scopolamine undergoes epoxide opening, and Riociguat has its electrophilic warhead in a group hydrolyzable carbamate. It is important to note that, according to the scope of our search, the beta-lactamase inhibitor Vaborbactam has not been proposed as an inhibitor against SARS-CoV-2 M^pro^, whereas Ixazomib has been proposed as a non-covalent inhibitor in computational approaches [50] and an analysis of the transcriptional response of the host to SARS-CoV-2 infection and the drug-sensitive gene relationship, highlighting the potential use of Ixazomib and Carfilzomib for its proteasome inhibitory activity (Oprozomib was also identified in this study) [51].

**Figure 5:**
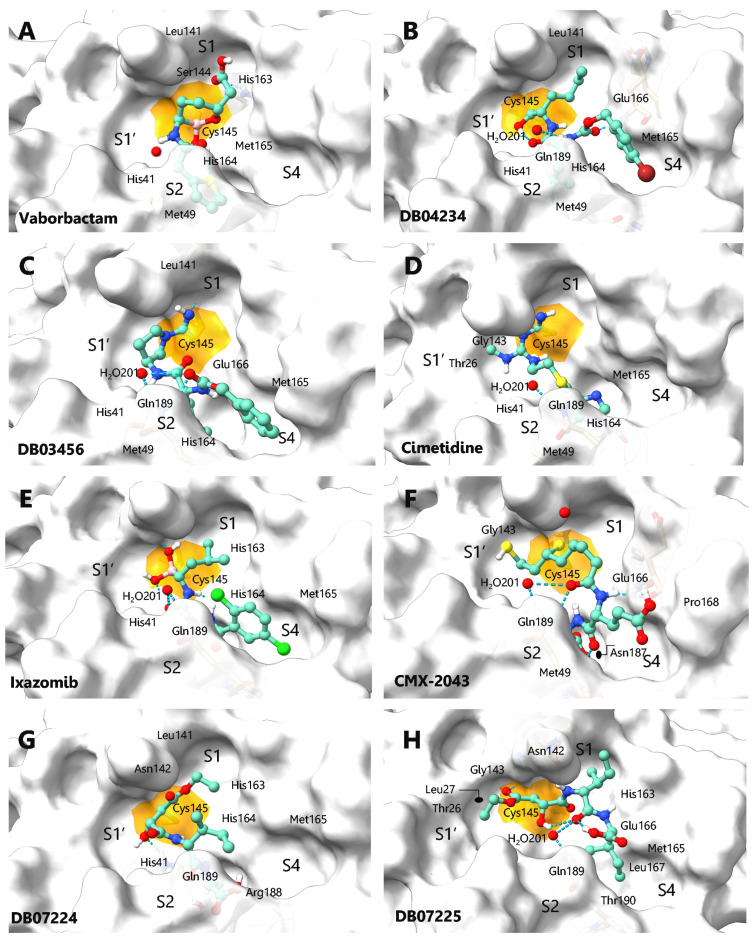

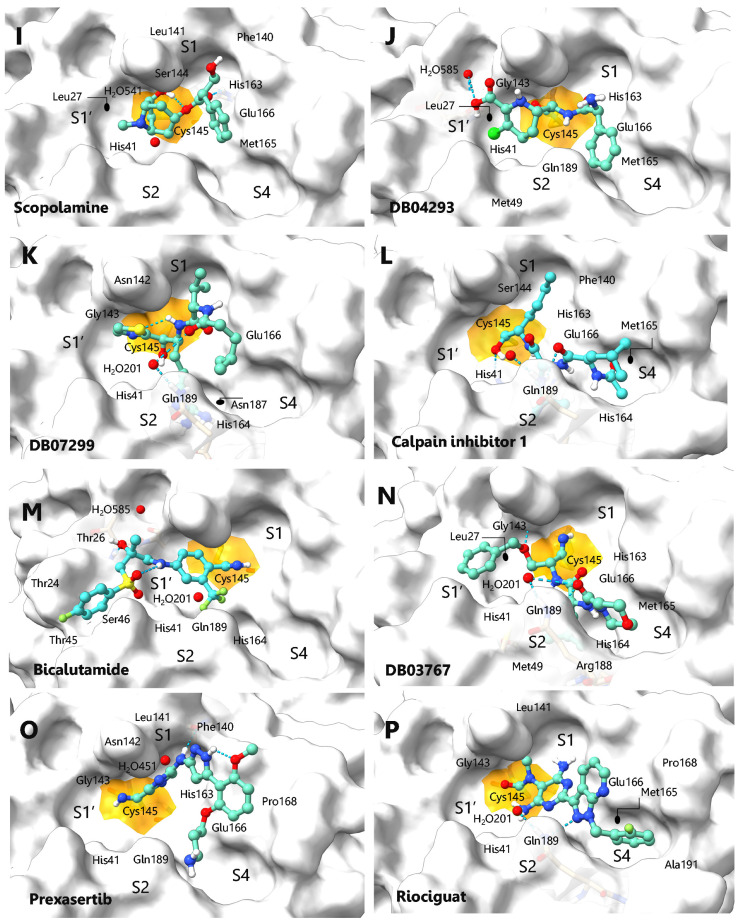
Binding pose of the top-ranked drugs after CsScore, (**A**) Vaborbactam, (**B**) DB04234, (**C**) DB03456, (**D**) Cimetidine, (**E**) Ixazomib, (**F**) CMX-2043, (**G**) DB07224, (**H**) DB07225, (**I**) Scopolamine, (**J**) DB04293, (**K**) DB07299, (**L**) Calpain inhibitor-1, (**M**) Bicalutamide, (**N**) DB03767, (**O**) Prexasertib, and (**P**) Riociguat. The covalent interaction generated by the Cys145 residue of M^pro^ with the electrophilic warhead of each molecule is shown in orange and the hydrogen bonding are represented in cyan segmented lines.

The binding modes of Vaborbactam and Ixazomib on M^pro^ showed a covalent inhibition of S1/S2 and S1/S2/S4, respectively (Figure 5A,E), and both organoborate drugs have the advantage of being able to generate covalent and reversible bonds with serine proteases in a clinically safe manner [52,53]. On the other hand, Cimetidine and Bicalutamide generated a covalent interaction with the corresponding carbonitrile group showing a different binding mode. The H2 antagonist showed molecular recognition in the central S1-S1′/S2 subpockets (Figure 5D), while the androgen receptor antagonist showed a completely different binding mode (Figure 5M), where the 4-fluorobenzenesulfonyl substituent was oriented beyond the S1′ subpocket, reaching hydrophobic interactions in a small cavity formed by Thr24, Thr45 and Ser46 (Figure S2), which until now has not been fully explored in the design of new inhibitors [54]. Experimental evidence indicates that histamine receptor antagonists can inhibit SARS-CoV-2 through the H1 receptor or the ACE2 receptor, and can also interrupt the interaction between heparan sulfate and the spike protein, slowing the entry of genetic material of SARS-CoV-2 [55]. On the other hand, it has been suggested that the combination of antagonists of the H1, H2 and H4 receptors is effective in reducing lung inflammation caused by SARS-CoV-2, reducing the severity of the infection [56]. Additionally, there is evidence of the covalent binding of Bicalutamide on M^pro^ reaching a partial inhibition at a concentration of 50 μM, data that corroborate our findings [57]. However, the most relevant application of prostate cancer agents such as Bicalutamide and Enzalutamide has to do with their ability to inhibit androgen signaling, reducing the expression of TMPRSS2 in the lung, and consequently preventing viral entry into human cells [58,59]. The last approved drug that appears on our list is Riociguat, which is used for the control of type IV pulmonary hypertension (secondary to chronic pulmonary embolism) and is considered the first stimulator of guanylate cyclase for clinical use. This drug showed a preferential binding mode of the S1/S4 type, prevailing hydrophobic interactions, and two acceptor hydrogen bond interactions between one of the nitrogen atoms of the pyrimidine nucleus and Glu166 as well as 1*H*-pyrazolo [3,4-*b*] pyridine and the amino acid Gln189, while the fluorobenzyl substituent was adequately positioned in the S4 cavity, generating hydrophobic interactions with Leu141, Met165, Pro168, and Ala191. Riociguat has not shown experimental efficacy against the virus; it has only been proposed against the spike protein fusion peptide with computational approaches [60].

Moving to the investigational drugs identified in our screening, it is striking that most of them are peptide-like structures except for DB04293 (carbacephem) and DB08614 (phenylpyridine). The 10 best-ranked drugs in this group have various electrophilic groups that can be consulted in Table S2; curiously, they have pharmacological activity on various proteases. For example, they act as cathepsin inhibitors such as DB04234, DB03456, DB07224, DB07225, Calpain inhibitor 1, and DB03767, beta-lactamase inhibitors DB04293 and Ceftaroline, coagulation factor XI inhibitor (DB07299), and a chymotrypsin-1 inhibitor elastase family member (DB08614).

The binding mode of DB04234 (Figure 5B) and DB03456 (Figure 5C) was of type S1/S2-S4, maintaining the interactions and similar energy data between both (entry B and C in Table 2); the difference lies in the electrophilic warheads corresponding to an aldehyde and nitrile group, respectively. CMX-2043 suffered the opening of the 1,2-dithiolane ring by nucleophilic attack by the Cys145 side chain, adopting a conformation that covers interactions in the S1-S1/S2-S4 regions, generating HDA interactions with the Glu166 and Pro168 residues, as well as HBD between the group -SH (generated by the opening of the ring) and Gly143 in addition to the nitrogen atom of an amide group with Glu166 (Figure 5F). Secondly, the ligands DB07224 and DB07225, despite maintaining a structural similarity with the same fit to the pharmacophoric map, showed some differences in their binding mode in M^pro^ (S1/S2 and S1-S1′/S2-S4, respectively). Although the binding energies in the catalytic pocket were similar, DB07225 increased the hydrophobic interactions according to its structural substituents. The carbacephem derivative DB04293 was coupled to M^pro^ through the opening of the β-lactam ring by the action of the nucleophilic amino acid Cys145, adopting a structural conformation that generated interactions in the S1-S1′/S2 sub-pockets (Figure 5J). β-lactam rings have shown good inhibition of SARS-CoV-2 M^pro^ in an in-vitro assay demonstrating covalent binding by mass spectrometry [61]; however, their clinical application remains defined for the treatment of complications of viral infection and is limited by probable resistance to antibiotics [62].

DB07299, the inhibitor of coagulation factor XI, showed interactions with the amino acids that make up the S1-S1′/S2 sub-pockets, forming a covalent anchor with Cys145 in the thiazole-2-carbonyl group (Figure 5K). In this case, it has been considered that the ketone groups are bioisosteric electrophilic centers with the cleavable amide carbonyl of the viral peptide substrates, for which they have been used successfully in the design of new selective inhibitors of viral protrusions, as is the case with the acyloxymethylketone and hydroxymethylketone derivatives developed by Pfizer that showed potent inhibitory activity and adequate pharmaceutical properties in preclinical studies [63,64]. In Figure 5N, the molecular recognition of DB03767 on M^pro^ can be observed, staying in the S1′/S2-S4 sites mainly, with evident polar interactions with the residues Gly146, Gln189, and a water molecule, while the hydrophobic interactions are generated with Leu27, His41, Met49, and Met165.

It is important to highlight the relevance that peptidomimetic M^pro^ inhibitors have acquired in the design of new antiviral agents; PF-07321332 (NCT04756531) was developed under this context, is considered a potent inhibitor of SARS-CoV-2 M^pro^, and has shown promising clinical results [65]. Structurally, it stands out for its electrophilic warhead, which corresponds to a carbonitrile group that favors maintaining good selectivity and a better toxicological profile, since this group has the characteristic of forming a reversible covalent interaction [66], a fact that gives confidence regarding the drugs identified in our screening.

Considering the above, cathepsin inhibitors have attracted powerful attention. Cathepsins are proteases with serine, cysteine, or aspartic acid residues as nucleophiles and are vital for digestion, coagulation, immune response, adipogenesis, hormone release, and peptide synthesis, among a wide variety of other functions [67]. Recently, cathepsin L was identified as a protease that elevates its surrounding concentration in COVID-19 patients and was positively correlated with the course and severity of the disease [68]. Additionally, it has been shown that Cathepsin L participates in the degradation of the extracellular matrix, an important process for SARS-CoV-2 to enter the host cell, and is even involved in the functional cleavage of the S protein, favoring the entry of the genetic material [69]. Cathepsins B, K, L, S, and V have also been shown to have proteolytic activity in various regions of the S protein [70]. Therefore, these types of enzymes that work in a proteolytic network are proposed as therapeutic targets to reduce the rate of viral infection. Even in this sense, inhibitors with Cathepsin/Mpro dual activity have been proposed, such as Calpain Inhibitor I and II [71], which were identified in our virtual screening data (Figure 5L, entry L in Table 2); recently, the dual activity of the inhibitor M-132 (Cbz-Leu-Leu-Leu-al) was also reported [72].

## 3. Materials and Methods

### 3.1. Drug Database

During the search for drugs with repositioning possibilities, in addition to considering the structural chemical constitution, it is relevant to consider the pharmacological and toxicological properties. Therefore, for this study, we used the FDA (https://www.fda.gov/drugs/drug-approvals-and-databases/, accessed on 25 March 2020) and DrugBank (https://www.drugbank.ca/releases/latest, accessed on 25 March 2020) databases, which are fed with approved drugs in clinical and experimental phases for various diseases. The files for each drug structure were obtained from the FDA database containing more than 1800 drugs and DrugBank with more than 9000. For our virtual screening analysis, the protocol to incorporate the set of molecules consisted of energy minimization using the Merck Molecular Force Field 94 (MMFF94s) calculation method for the 3D structure. Subsequently, the structural conformational search of each molecule was carried out using, in both cases, the iCon tool in LigandScout 4.4 Advance, maintaining the best search conditions (the maximum number of conformations was 300, and the RMS threshold was 0.8 Å, discarding duplicate conformations) [73].

### 3.2. Structure-Based (SB) Pharmacophore Model

A 3D structure-based pharmacophore model was built from the crystalline protein–ligand complex of the main protease of SARS-CoV-2 and the inhibitor N3 (7BQY), obtained from the PDB protein database (https://www.rcsb.org/, accessed on 8 June 2020), with a resolution of 1.7 Å [28]. The binding site of the crystalline complex was identified, and its minimization energy was calculated using Merck Molecular Force Field 94 (MMFF94s) considering a solvated environment. Subsequently, the pharmacophore model was created using the pharmacophore generation tool of the LigandScout 4.4 Advanced software. The pharmacophore model provided the 3D coordinates of the minimal molecular interactions of the co-crystallized inhibitor (N3) binding mode at the M^pro^ recognition site of the virus.

### 3.3. Pharmacophore Model-Based Virtual Screening

Structure-based virtual screening was carried out in LigandScout 4.4 Advanced considering the coordinates and distance (Å) between the pharmacophoric features obtained from the 7BQY complex. Pharmacophore model-based virtual screening was described as an efficient virtual detection tool that defines the spatial relationship between the pharmacophoric features that represent the interaction properties between the receptor and the ligand. The tolerance spheres of the pharmacophoric characteristics were 1.50 Å, except for the covalent sphere, which was 1.95 Å; the screening mode used was “match all query features”, and the scoring function was determined by the percentage of “pharmacophore-fit”. The search by molecule was continued until the “best match conformation” was obtained considering the exclusion volumes [74]. The reliability of the screening was validated with the reference inhibitor N3 for the protein (7BQY). The energies (Kcal/mol) of binding enthalpy, complex energy, and binding affinity were calculated from the best-scored molecules. The final selection was based on visual inspection of the highest-ranking compound docking poses, complemented by the best affinity values obtained with the Autodock Vina 1.1 extension set in LigandScout 4.4 Advanced.

### 3.4. Covalent Docking

The covalent docking was performed with Molecular Operating Environment (MOE) software version 2019.0102. The DOCK module of MOE achieved conformational sampling by placement methodology [75]. Before the docking studies, each compound undergoes energy minimization and the atomic charges are adjusted, followed by a potential energy adaptation, using MMFF94s force field. The protein 7BQY was prepared using default parameters (generating protonation states by default, pH = 7 ± 2), the co-crystallized ligand was removed from the active site, hydrogen atoms were added over the selected receptor, and then the potential energy was fixed. The final energy, induced-fit docking score, was evaluated using the GBVI/WSA ΔG scoring function with the Generalized Born solvation model (GBVI) [76]. The GBVI/WSA ΔG is a forcefield-based scoring function [77], which estimates the free energy of binding of the ligand from a given pose. It has been trained using the MMFF94x and AMBER99 forcefields on the 99 protein–ligand complexes of the solvated interaction energy (SIE) training set [78]. All of the ligands of the molecular database were tested according to the above procedure. The Amber12:EHT force field was used for all computational procedures. Cys145 was selected as a reactive residue (nucleophilic group at -SH) in the catalytic site of SARS-CoV-2 M^pro^. The type of covalent reaction was identified from the interaction predicted in the pharmacophoric hypothesis generated between the sulfur atom located in the side chain of Cys145 and the electrophilic center of the drugs selected in the structure-based virtual screening, considering a nucleophilic addition reaction. The binding mode and chemical stability generated by the ligands at the catalytic site allow for successful covalent coupling, in addition to changes in charge, bond order, protonation states, and the stereochemistry of the reacting species. The pose prediction is generated by the reaction or transformation methodology of the combinatorial library. The covalent interactions identified in the virtual screening on the pharmacophoric map that was not included in the catalog of predefined reactions in MOE were customized using the ChemDraw Professional 15.0 software. The visualization of the docking results was performed with ChimeraX (https://www.cgl.ucsf.edu/chimerax/, accessed on 25 March 2019).

Finally, the selection of molecular hits was performed under a consensus score (CsS) calculated with the following equation: CsS = E_C_ − E_BE_ + [(|∆G_ncov_| + |∆G_cov_|) (S_PF_/100)]. Ec represents the general energy of the complex and E_BE_ refers to the binding enthalpy energy (the lower value of the enthalpy of binding represents an energetically more favorable environment for the interaction between a drug and the recognition site within the protein). Subsequently, the non-covalent (∆G_ncov_) and covalent (∆G_cov_) docking energies were added, multiplying the quotient SPF/100, which represents the number of minimal pharmacophoric interactions to achieve molecular recognition and covalent inhibition.

## 4. Conclusions

**Figure 6:**
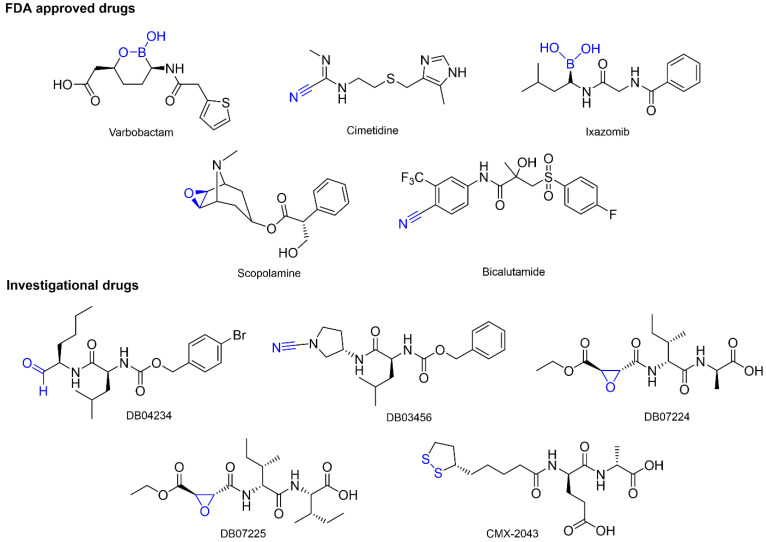
Top five FDA drugs approved and the top five investigational drugs, obtained by a combined structure-based strategy of the pharmacophoric model, virtual screening, and covalent docking. The electrophilic warhead of the promising drugs is shown in blue.

The health emergency due to COVID-19 requires urgent treatments to stop and prevent viral infection; in this context, bioinformatics approaches provide relevant information for the design of antiviral drugs and even vaccines. An important area of application is the identification of new ligands through structure-based high-throughput virtual screening. In this work, we used 3D pharmacophore models that allowed us to precisely define the minimum molecular interactions that a ligand requires to covalently bind to SARS-CoV-2 M^pro^ in combination with conventional and covalent docking tools, potential non-peptide and peptide-like M^pro^ inhibitors were identified (Figure 6). Our combined structure-based virtual screening strategy showed high efficiency and reliability in identifying FDA-approved drugs (Vaborbactam, Cimetidine, Ixazomib, Scopolamine, Bicalutamide, and Riociguat) and protease inhibitor drugs as potential irreversible M^pro^ inhibitors. The certainty of identification was based on the ability of the set of tools to correlate drugs that have experimentally shown anti-COVID activity via inhibition of M^pro^, increasing the probability of success of the drugs proposed here in future in-vitro assays and managing to provide useful information on the current status of the design and discovery of covalent SARS-CoV-2 M^pro^ and potential dual-activity Cathepsin/M^pro^ inhibitors.

Our work demonstrates a combined cheminformatics procedure that can predict the structure and affinity of effective molecules for a challenging drug target (such as SARS-CoV-2 M^pro^), through a set of steric and electronic features that are necessary to ensure supramolecular interactions optimal with the biological objective. This combined approach provides an efficient strategy for the discovery and repositioning of drugs that can target different proteins of viruses and other potential pathogens.

## Figures and Tables

**Table 1:** Top-ranked DrugBank and FDA drugs from structure-based virtual screening of SARS-CoV-2 M^pro^.

Drug	Interactions *^a^*													S_PF_ *^b^*	E_BE_ *^c^*	E_BS_ *^d^*	E_C_ *^e^*	S_BA_ *^f^*	ΔG_ncov_ *^g^*
	T25	M49	C145		H164	M165	E166		L167	Q189	T190	A191	H_2_0						
INHIBITOR N3	H	H	HBA	CI	HBD	H	HBD	HBA	H	HBD	HBD	H	HBA	106.93	5034.60	713.40	5815.39	−34.11	−7.70
INHIBITOR N1		H	HBA	CI	HBD	H	HBD	HBA	H	HBD	HBD	H	HBA	106.90	3132.36	642.09	3819.69	−21.22	−6.80
INHIBITOR N9		H		CI	HBD	H	HBD	HBA	H	HBD	HBD		HBA	96.23	2216.40	540.26	2796.46	−15.40	−6.30
INHIBITOR I2		H		CI	HBD	H	HBD	HBA	H	HBD			HBA	86.48	1112.63	617.64	1770.67	−9.61	−7.00
IXAZOMIB		H		CI	HBD	H	HBD	HBA	H	HBD			HBA	83.90	170.89	469.81	674.98	−3.22	−6.70
CALPAIN INH		H		CI	HBD		HBD	HBA		HBD			HBA	77.81	630.46	457.86	1127.00	−22.19	−5.10
DB08119		H	HBA	CI	HBD			HBA		HBD			HBA	77.80	2071.81	491.17	2619.91	−27.30	−6.20
DB03984		H	HBA	CI	HBD			HBA		HBD			HBA	76.82	2337.41	522.87	2930.08	−21.93	−6.70
DB07224		H	HBA	CI	HBD			HBA		HBD			HBA	76.48	156.99	476.63	659.17	−15.30	−6.10
DB07225	H	H	HBA	CI	HBD			HBA		HBD			HBA	76.48	204.10	479.96	705.99	−1.17	−5.80
AMPRENAVIR		H	HBA	CI	HBD	H			H	HBD			HBA	76.23	679.32	593.57	1324.18	−20.21	−7.80
OPROZOMIB		H		CI			HBD	HBA		HBD	HBD		HBA	76.12	2033.90	577.90	2696.86	−19.37	−6.30
CARFILZOMIB		H		CI	HBD		HBD	HBA		HBD			HBA	76.03	5948.19	756.99	6849.93	−36.01	−8.20
DB03456		H	HBA	CI	HBD			HBA		HBD			HBA	75.87	103.11	461.78	584.84	−14.91	−6.80
DB07299		H		CI	HBD	H	HBD	HBA	H				HBA	75.65	398.77	580.32	934.01	2.54	−6.50
DB03767		H	HBA	CI	HBD			HBA		HBD			HBA	75.52	504.95	523.68	1074.14	−18.57	−6.60
DB04234	H	H		CI	HBD			HBA		HBD			HBA	75.34	62.13	457.82	544.49	−3.05	−6.10
BOCEPREVIR		H		CI	HBD	H	HBD		H				HBA	75.28	266.36	474.85	824.16	−9.45	−5.40
VABORBACTAM			HBA	CI	HBD	H		HBA	H	HBD			HBA	73.71	−10.58	457.61	453.04	−6.40	−6.50
DB07749		H		CI	HBD			HBA		HBD			HBA	67.84	2577.53	485.06	3106.40	−17.85	−6.10
CEFTAROLINE	H	H	HBA	CI			HBD	HBA					HBA	66.62	2556.58	698.58	3252.33	17.64	−7.10
TELAPREVIR				CI	HBD		HBD	HBA			HBD		HBA	66.43	4479.26	576.38	5196.13	−21.93	−6.20
DB07160		H	HBA	CI	HBD								HBA	66.40	924.43	487.56	1420.27	−19.95	−5.20
PREDNISONE		H	HBA	CI			HBD			HBD			HBA	66.34	1095.73	551.05	1738.53	−3.08	−7.30
DARUNAVIR		H	HBA	CI	HBD	H		HBA	H	HBD			HBA	66.32	340.83	471.19	861.32	−30.86	−7.50
PREXASERTIB			HBA	CI		H		HBA	H	HBD			HBA	66.27	339.96	514.87	921.30	−16.68	−7.50
TEMOCILLIN		H	HBA	CI	HBD			HBA					HBA	66.11	1131.23	475.67	1660.75	−11.36	−6.80
CIMETIDINE				CI	HBD	H		HBA	H		HBD		HBA	65.99	120.44	459.12	557.49	−10.99	−5.30
SCOPOLAMINE		H	HBA	CI				HBA		HBD			HBA	65.98	122.54	459.65	646.78	−6.35	−6.30
FLUOXYMESTERONE		H	HBA	CI				HBA		HBD			HBA	65.98	1110.31	519.19	1733.89	−20.21	−7.20
METHSCOPOLAMINE		H	HBA	CI				HBA		HBD			HBA	65.98	1476.45	510.42	2070.99	−17.74	−7.00
DB08614		H		CI	HBD		HBD	HBA					HBA	65.93	623.04	492.22	1151.02	−19.01	−7.30
FLOVAGATRAN				CI	HBD	H	HBD	HBA	H				HBA	65.86	1325.16	516.40	1892.44	−7.04	−7.00
FELYPRESSIN		H	HBA	CI		H	HBD		H				HBA	65.83	9699.06	993.86	10,838.66	−19.45	−6.80
DB07987		H		CI	HBD			HBA		HBD	HBD			65.72	1777.82	554.85	2342.50	−18.83	−6.00
AZTREONAM			HBA	CI	HBD		HBD						HBA	65.62	944.90	570.26	1482.14	5.99	−6.80
PREDNISOLONE		H	HBA	CI			HBD			HBD			HBA	65.59	1507.06	506.38	2120.64	−16.09	−7.80
RIOCIGUAT		H	HBA	CI	HBD	H			H				HBA	65.57	348.80	529.93	955.36	−10.18	−8.30
TIOTROPIUM		H	HBA	CI				HBA		HBD			HBA	65.57	858.19	513.34	1466.31	−7.52	−7.00
CMX−2043			HBA	CI		H	HBD	HBA	H	HBD				65.45	156.96	472.39	634.24	−13.38	−5.90
GAXILOSE				CI	HBD		HBD	HBA		HBD			HBA	65.45	2334.44	578.92	2999.85	15.21	−6.50
CINOLAZEPAM		H	HBA	CI				HBA		HBD			HBA	65.40	2514.84	846.67	3451.69	−15.19	−7.40
Mdl 101,146		H	HBA	CI		H		HBA	H				HBA	65.33	4065.30	624.83	4813.17	−16.43	−7.20
FOSAMPRENAVIR		H	HBA	CI	HBD	H			H				HBA	65.33	6894.18	648.29	7505.59	−10.23	−7.20
BICALUTAMIDE		H	HBA	CI			HBD	HBA		HBD				65.19	376.99	497.65	932.83	−15.10	−7.40
DB04293	H	H	HBA	CI	HBD								HBA	65.17	152.09	483.24	704.77	0.79	−7.30
ATAZANAVIR		H		CI	HBD		HBD	HBA					HBA	65.17	7923.60	860.74	8900.26	−28.37	−5.40
BMS−488043	H	H	HBA	CI	HBD			HBA					HBA	65.15	1800.92	537.19	2446.41	−6.45	−7.90
DB04232	H	H	HBA	CI	HBD								HBA	65.10	1768.70	529.64	2367.43	−1.12	−6.90
CEPHALOGLYCIN		H	HBA	CI	HBD					HBD			HBA	64.48	3432.94	596.57	4091.77	−8.54	−7.60
MUPIROCIN		H	HBA	CI				HBA		HBD			HBA	63.87	4899.57	820.50	5761.68	−13.58	−6.70
CEFDITOREN	H	H		CI	HBD			HBA					HBA	63.78	3465.77	799.57	4352.81	−3.02	−6.50
CABAZITAXEL		H		CI	HBD	H		HBA	H		HBD		HBA	63.67	7750.80	1172.22	9132.50	−23.63	−6.70

*^a^* Matching feature with the pharmacophoric hypothesis structure-based PDB ID: 7BQY complex. Hydrogen bonding acceptor (HBA, red), hydrogen bonding donor (HBD, green), hydrophobic interaction (H, yellow) and covalent interaction (CI, orange). The interactions between the functional groups of each drug with the amino acids can be consulted in Table S1. *^b^* **S_PF_** = Pharmacophore-fit score, Fit value indicates how well the features in the pharmacophore map with the chemical features present in the compound. *^c^* **E_BE_** = MMFF94 Binding enthalpy, is the contribution of the calculated strain energy of the ligand in its active conformation, the energy of the geometric optimization of a relaxed conformation and the energy of protein-ligand interaction (represented by the sum of intermolecular Coulomb and Van der Waals terms). *^d^* **E_BS_** = The binding site energy is the contribution of each residue of the ligand-protein interaction. *^e^* **E_C_** = Complex energy. *^f^* **S_BA_** = Calculated binding affinity score between the library molecules and the macromolecule, helps to gain an understanding of how well a ligand binds in the current environment. *^g^* Affinity energy calculated in Autodock Vina 1.1.

**Table 2:** Top-ranked drugs by the consensus score. The values of pharmacophore fit and affinity energies for docking are shown, as well as the relevant amino acids in molecular recognition.

Drug		CsScore	SPF ^*a*^	ΔGncov ^*b*^	ΔGcov ^*c*^	Interactions
Ref	INHIBITOR N3	412.82	106.93	−7.7	−10.1	Leu27, His41, Met49, Asn142, Gly143, Ser144, Cys145, His164, Met165, Glu166, Leu167, Pro168, Gln 189, Thr190, Ala191, H_2_O201
(A)	VABORBACTAM	436.08	73.71	−6.5	−5.9	His41, Met 49, Leu141, Ser144, Cys145, His163, His164, Met165
(B)	DB04234	487.59	75.34	−6.1	−7.3	His41, Met 49, Leu141, Cys145, His164, Met165, Glu166, Gln 189, H_2_O201
(C)	DB03456	496.79	75.87	−6.8	−6.1	His41, Met 49, Leu141, Cys145, His164, Met165, Glu166, Gln189, H_2_O201
(D)	CIMETIDINE	503.00	65.99	−5.3	−5.3	Thr26, His41, Met49, Gly143, Cys145, His164, Met165, Gln189, H_2_O201
(E)	IXAZOMIB	521.07	83.90	−6.7	−5.9	His41, Cys145, His163, His164, Met165, Leu167, Gln189, Thr190, H_2_O201
(F)	CMX-2043	523.19	65.45	−5.9	−6.8	His41, Met 49, Gly143, Glu166, Pro168, Asp187, Gln189, H_2_O201
(G)	DB07224	529.37	76.48	−6.1	−6.6	His41, Leu141, Asn142, Cys145, His163, His164, Met165, Arg188, Gln189, H_2_O201
(H)	DB07225	540.42	76.48	−5.8	−6.6	Leu27, Thr26, Asn142, Gly143, Cys145, His163, Met165, Glu166, Leu167, Thr190
(I)	SCOPOLAMINE	558.11	65.98	−6.3	−5.6	Leu27, His41, Phe140, Leu141, Ser144, Cys145, His163, Met165, Glu166, H_2_O541
(J)	DB04293	596.86	65.17	−7.3	−6.2	Thr26, His41, Met49, Gly143, Cys145, His163, Met165, Glu166, Gln189, H_2_O585.
(K)	DB07299	621.90	75.65	−6.5	−7.3	His41, Asn142, Gly143, Cys145, His164, Met165, Glu166, Asp187, Gln189, H_2_O201
(L)	CALPAIN INH-1	626.71	77.81	−5.1	−7.4	His41, Phe140, Ser144, Cys145, His163, His164, Met165, Glu166, Gln189
(M)	BICALUTAMIDE	678.69	65.19	−7.4	−5.5	Thr24, Thr26, His41, Thr45, Ser46, Cys145, His164, Gln189, H2O201, H_2_O585
(N)	DB03767	682.37	75.52	−6.6	−7.2	Leu27, His41, Met49, Gly143, Cys145, His163, His164, Met165, Glu166, Arg188, Gln189, H_2_O201
(O)	PREXASERTIB	687.56	66.27	−7.5	−5.3	His41, Phe140, Leu141, Asn142, Gly143, Cys145, His163, Glu166, Pro168, Gln189 H_2_O541
(P)	RIOCIGUAT	717.46	65.57	−8.3	−5.7	His41, Leu141, Gly143, Cys145, Met165, Glu166, Pro168, Gln189, Ala191, H_2_O201

*^a^***S_PF_** = Pharmacophore-fit score, Fit value indicates how well the features in the pharmacophore map with the chemical features present in the compound. *^b^* **ΔG_ncov_** = Affinity energy calculated in Autodock Vina 1.1.; *^c^* **ΔG_cov_** = Affinity energy calculated by covalent docking.

## Data Availability

The data presented in this study are available on request from the corresponding authors.

## Supplementary Materials

The following are available online at https://www.mdpi.com/article/10.3390/ijms23073987/s1.
