# Supplementary material for: In Silico Drug Repositioning to Target the SARS-CoV-2 Main Protease as Covalent Inhibitors Employing a Combined Structure-Based Virtual Screening Strategy of Pharmacophore Models and Covalent Docking
Source: Int J Mol Sci. 2022 Apr 3;23(7):3987. doi: 10.3390/ijms23073987 (PMC8999907; doi:10.3390/ijms23073987)
Supplement: Supplementary file 1 [file ijms-23-03987-s001.zip › ijms-1597914-supplementary.pdf]

In silico drug repositioning to target the SARS-CoV-2 main protease as covalent inhibitors employing a combined structure-based virtual screening strategy of pharmacophore models and covalent docking.

Luis Heriberto Vázquez Mendoza[1], Humberto L. Mendoza-Figueroa[1, *], Juan Benjamín García-Vázquez[1, 2, **], José Correa-Basurto[1], and Jazmín García-Machorro[3].

1 Laboratorio de Diseño y Desarrollo de Nuevos Fármacos e Innovación Biotecnológica (Laboratory for the Design and Development of New Drugs and Biotechnological Innovation), Posgrado en Farmacología de la Escuela Superior de Medicina del Instituto Politécnico Nacional. Plan de San Luis y Salvador Díaz Mirón s/n, Casco de Santo Tomás, Ciudad de México 11340. Mexico.

2 Cátedras CONACyT

3 Laboratorio de Medicina de la Conservación, Escuela Superior de Medicina del Instituto Politécnico Nacional. Plan de San Luis y Salvador Díaz Mirón s/n, Casco de Santo Tomás, Ciudad de México 11340. Mexico.

* Correspondence: H.L.M.F. hmendozaf@ipn.mx ** Co-correspondence: J.B.G.V. benjagv_5202@hotmail.com

Table S1. 2D representation of the interactions of the top-ranked drugs (pharmacophore fit >60) on the virtual screening on the pharmacophoric map based on the structure of SARS-Cov-2 M^{pro}/N3.

**Inhibitor N3**	**Inhibitor N1**
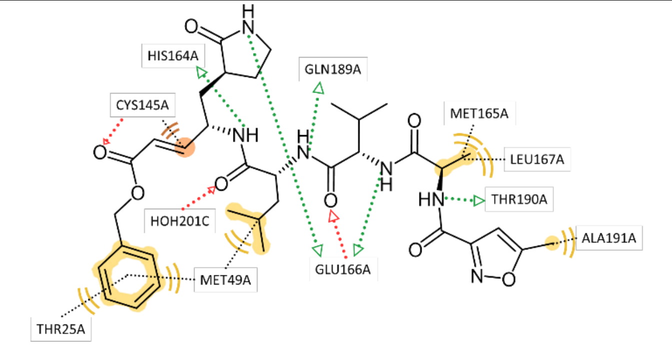	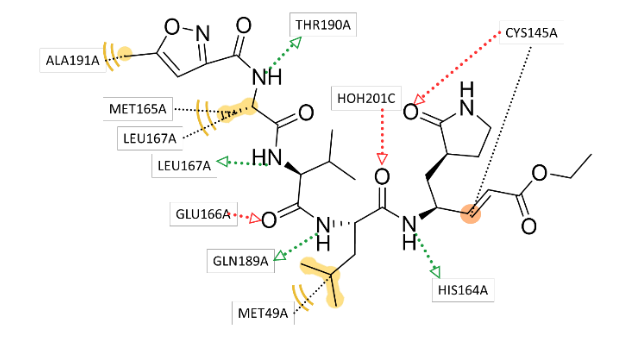
**Inhibitor N9**	**Inhibitor I2**
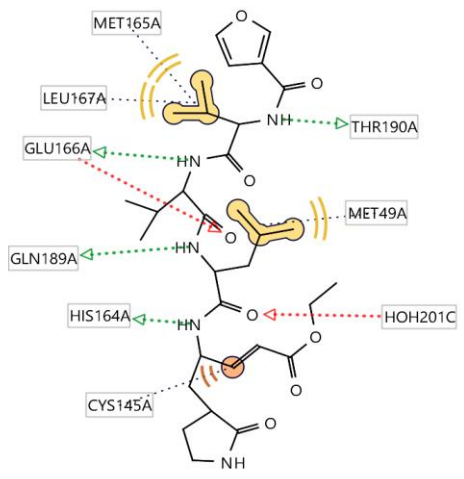	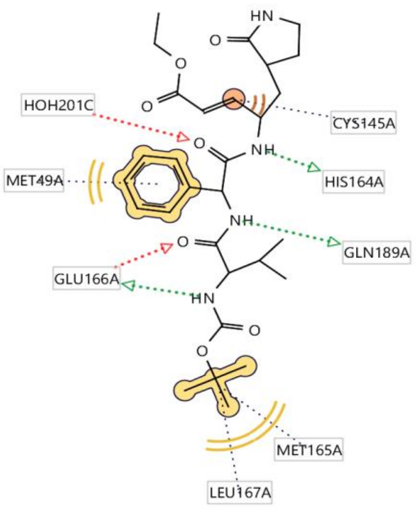
**Ixazomib**	**Calpain inhibitor I**
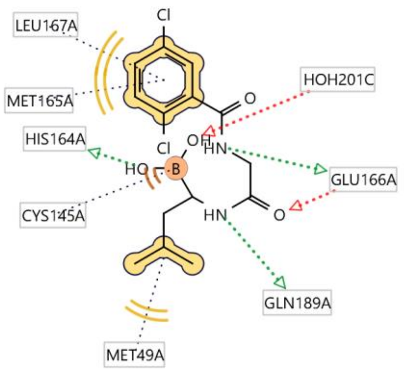	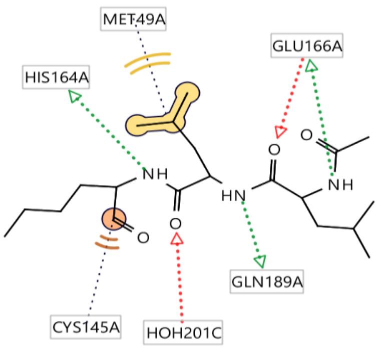

DB08119	DB03984
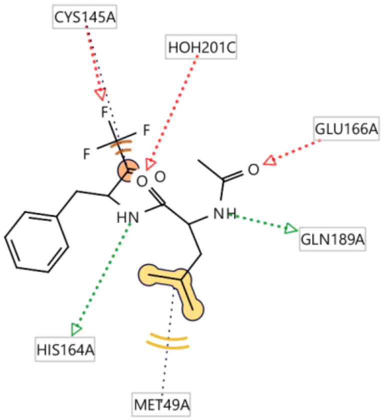	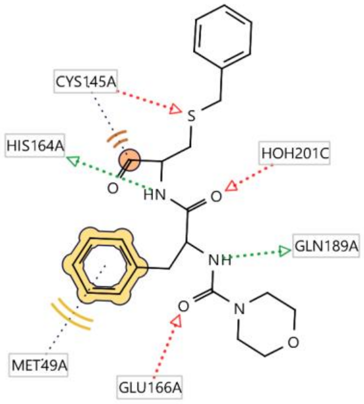

DB07224	DB07225
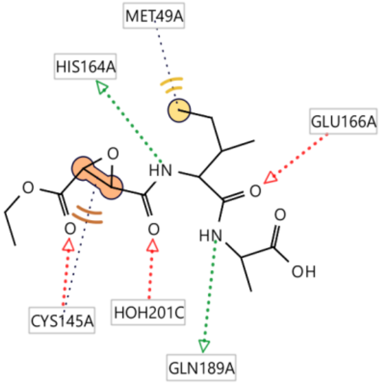	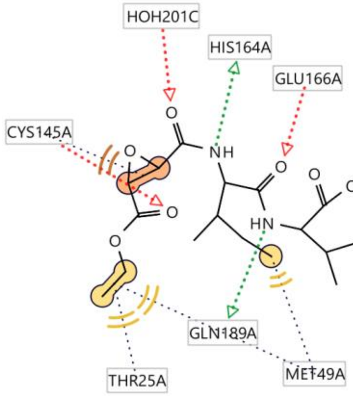

Amprenavir	Oprozomib
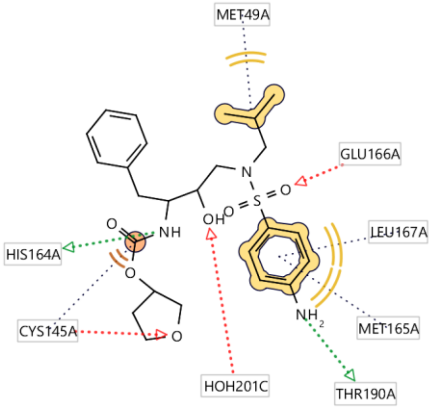	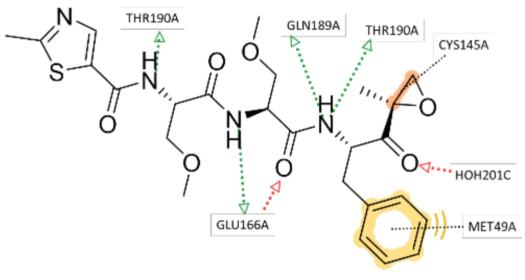

Carfilzomib

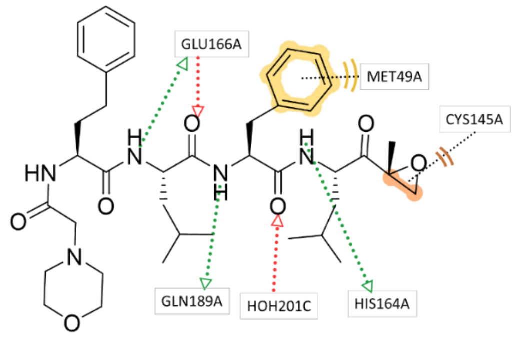

DB07299

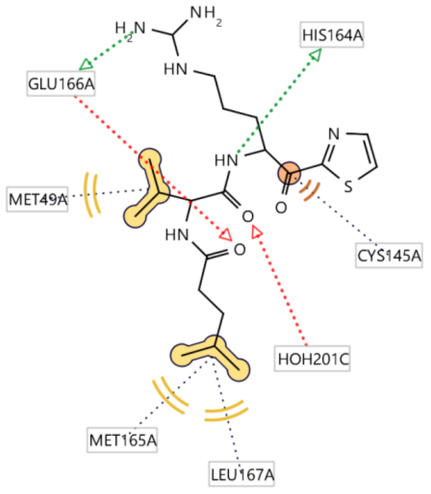

DB03767

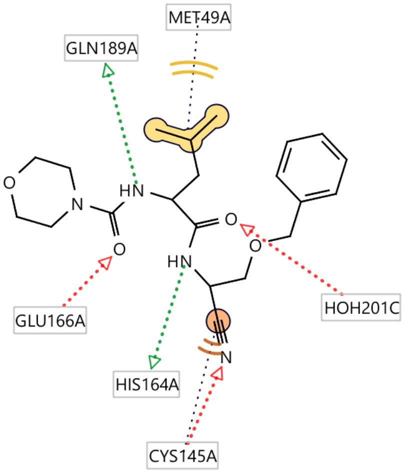

DB04234

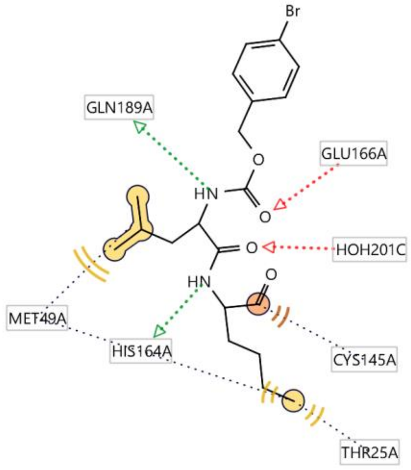

Boceprevir

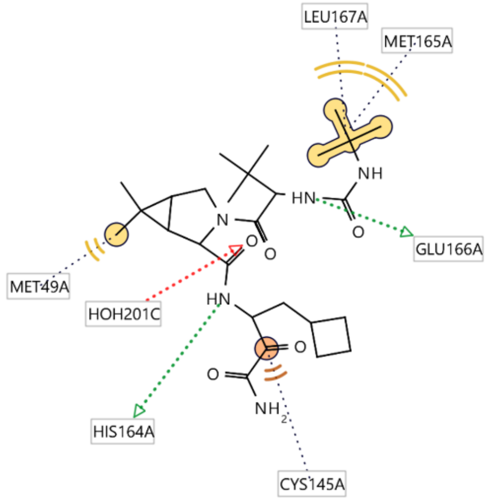

Vaborbactam

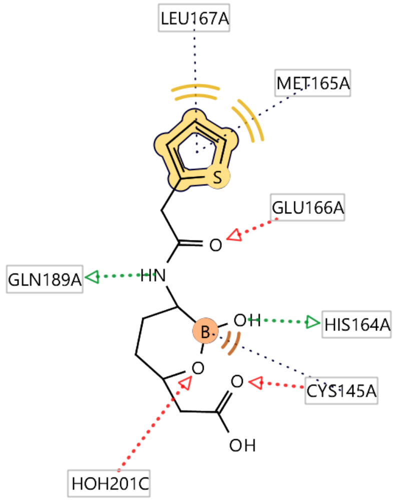

DB07749

Ceftaroline

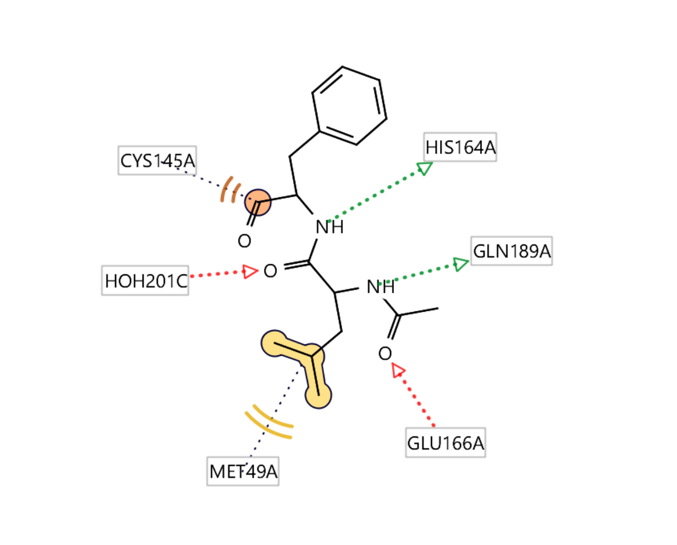

Telaprevir

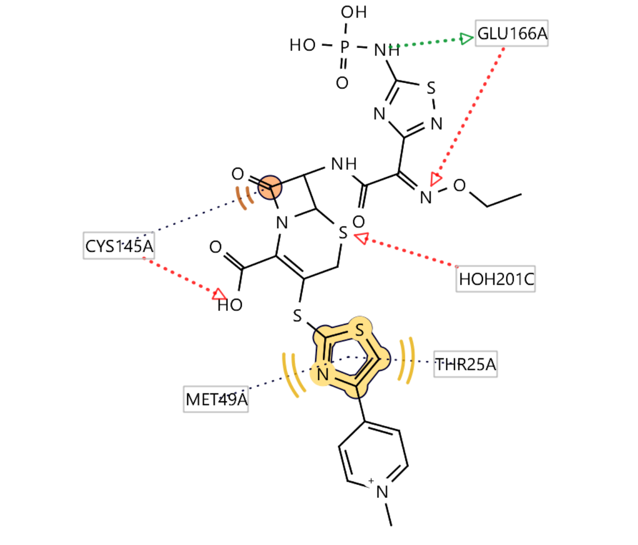

DB07160

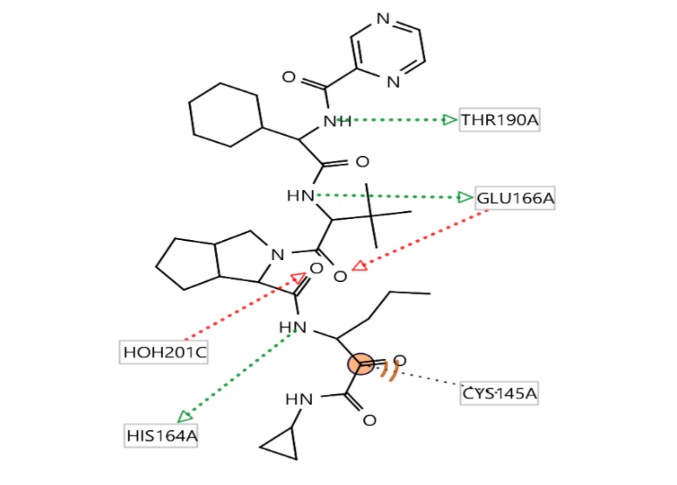

Prednisone

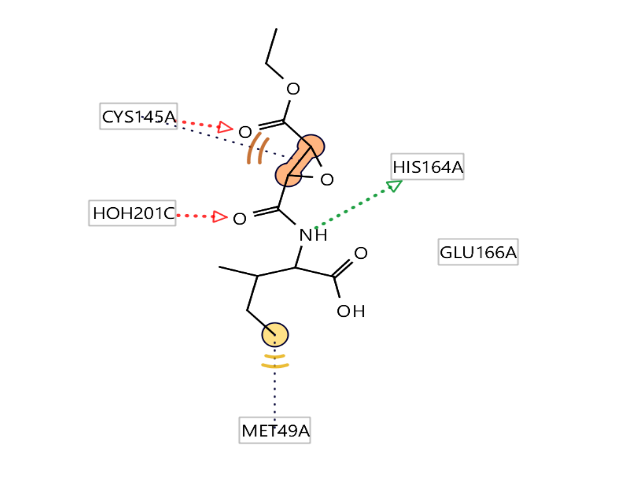

Darunavir

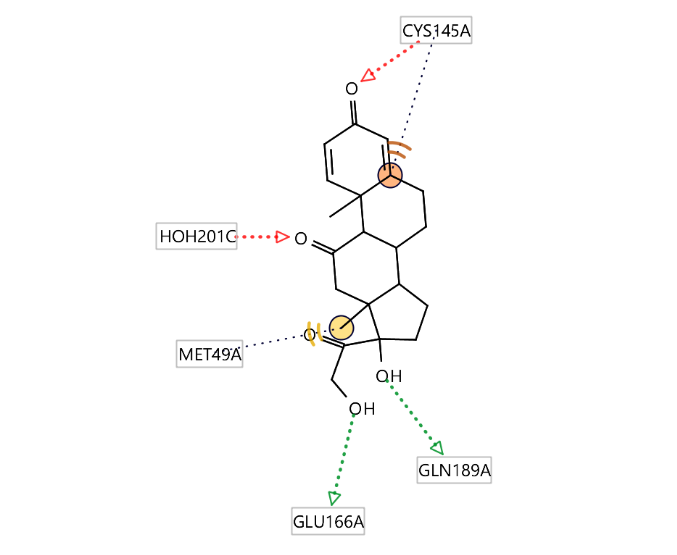

Prexasertib

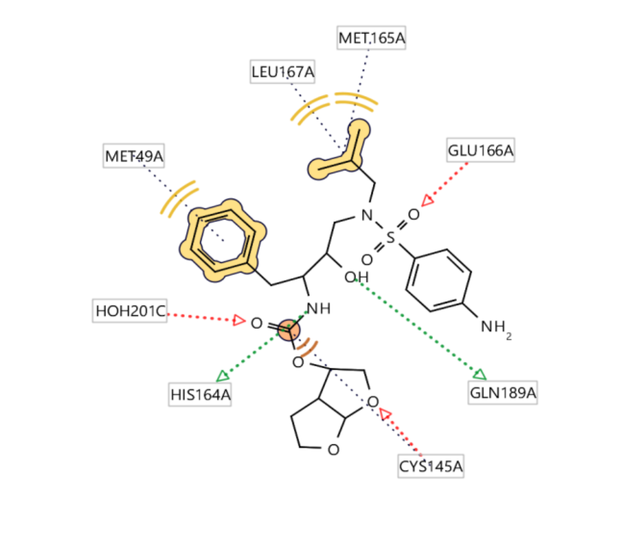

Temocillin

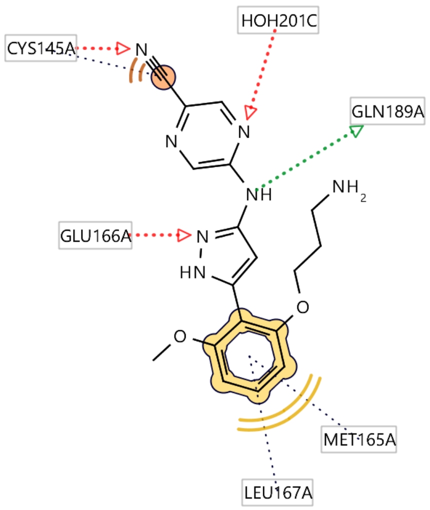

Cimetidine

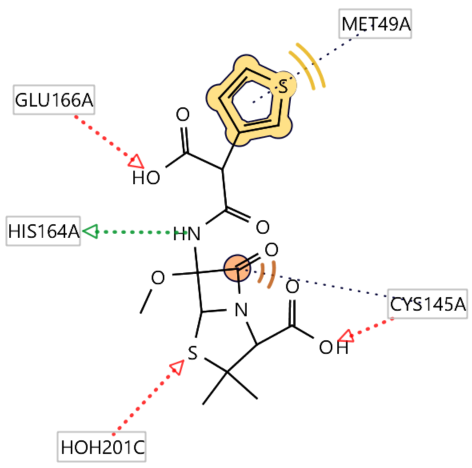

Scopolamine

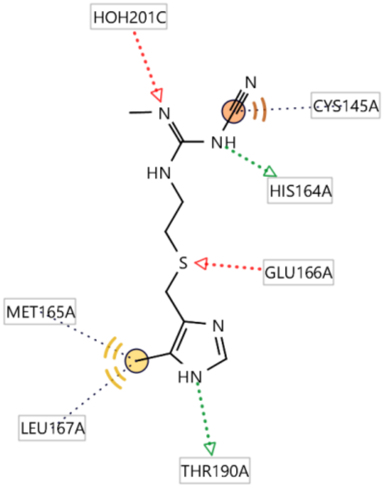

Fluoxymesterone

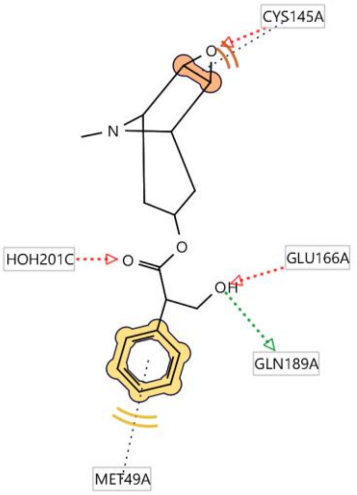

Methscopolamine

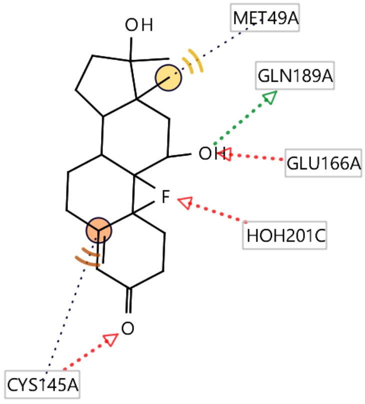

DB08614

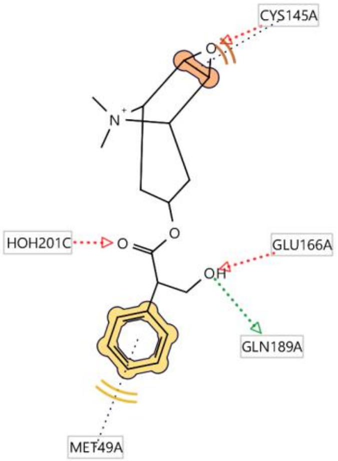

Flovagatran

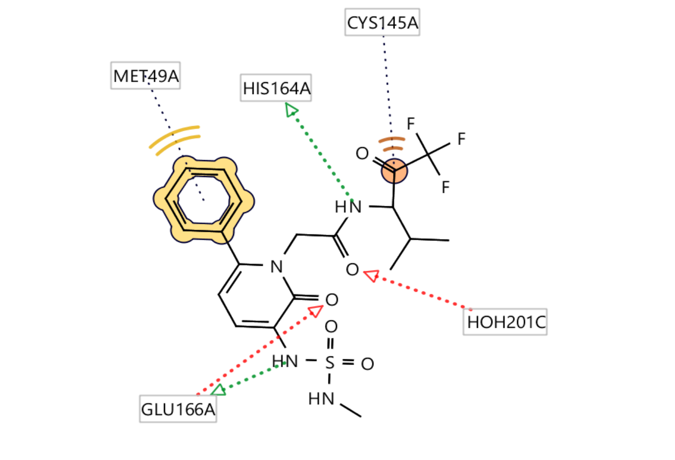

Felypressin

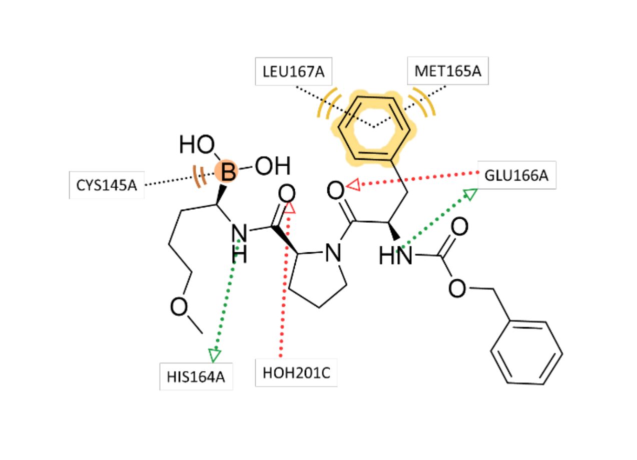

DB07987

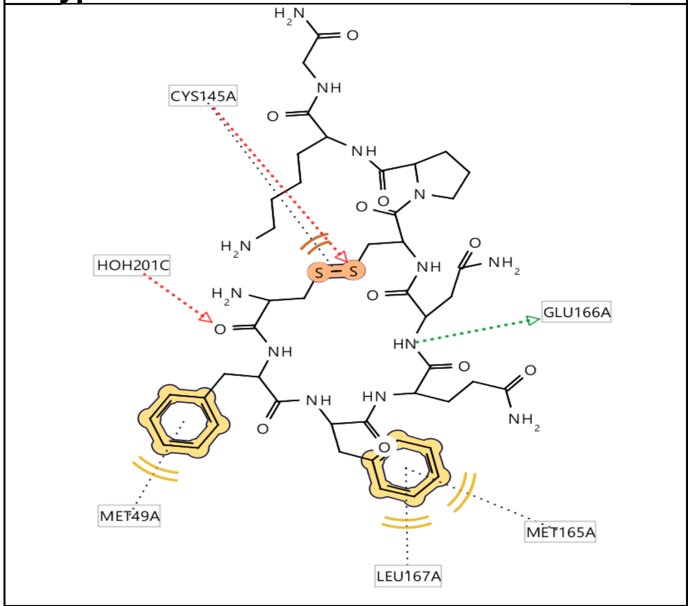

Aztreonam

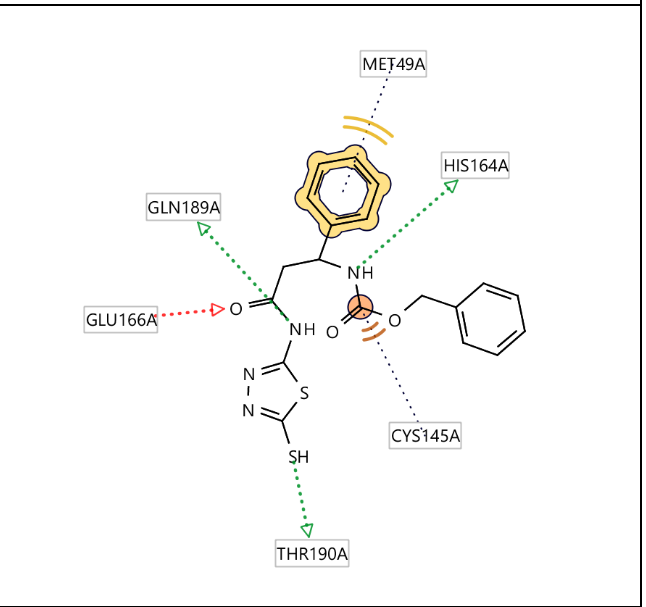

Prednisolone

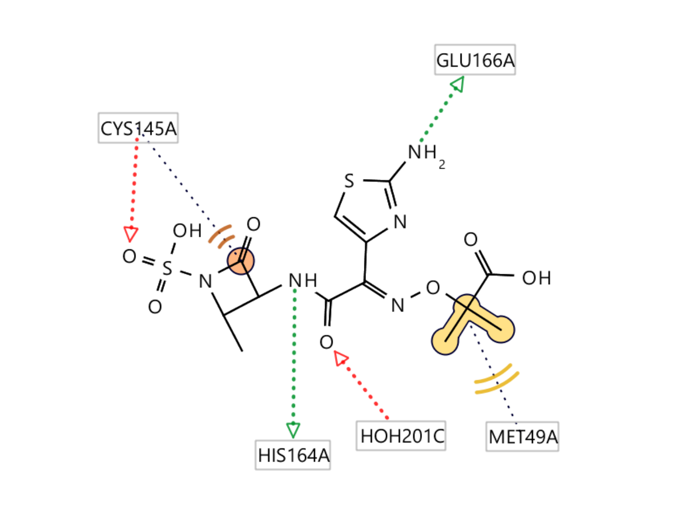

Riociguat

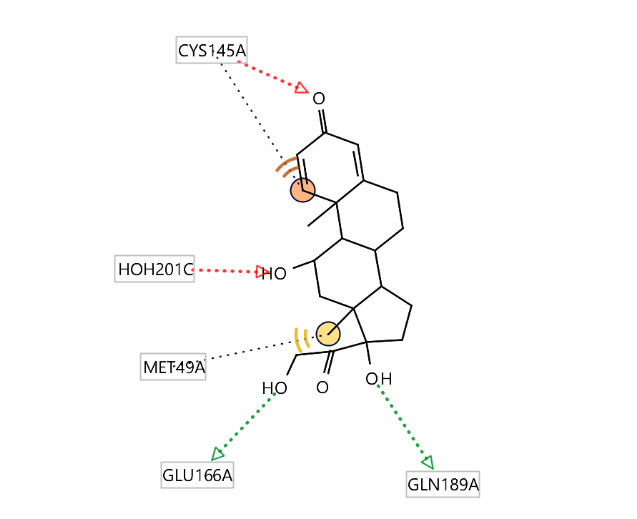

Tiotropium

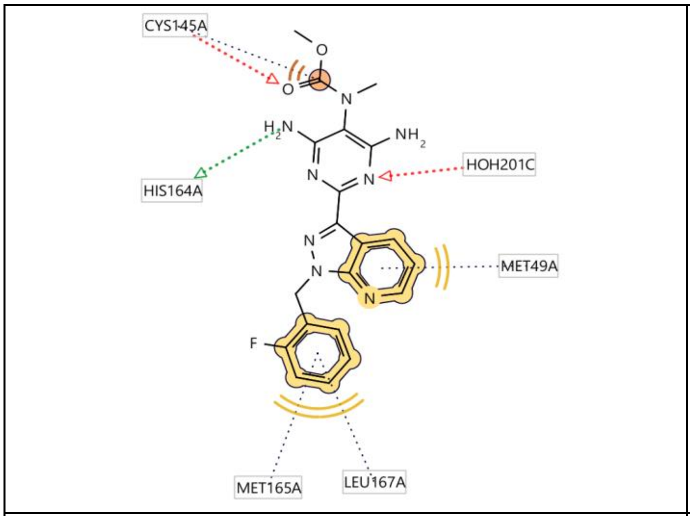

CMX-2043

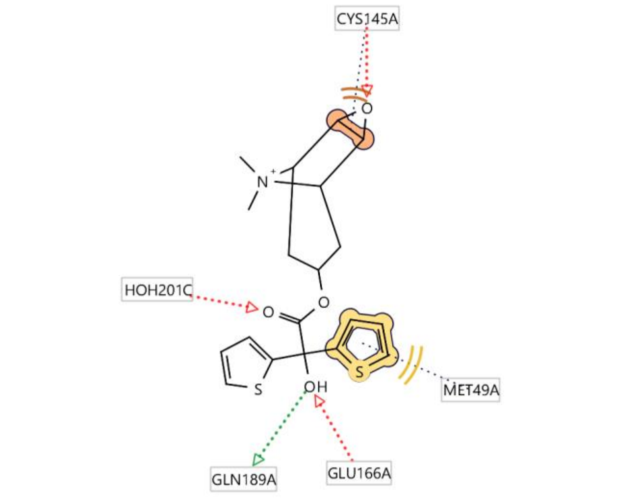

Gaxilose

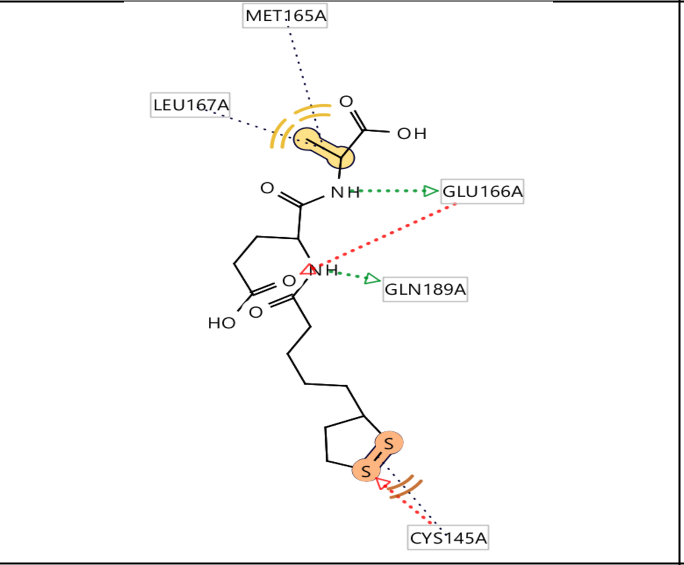

Cinolazepam

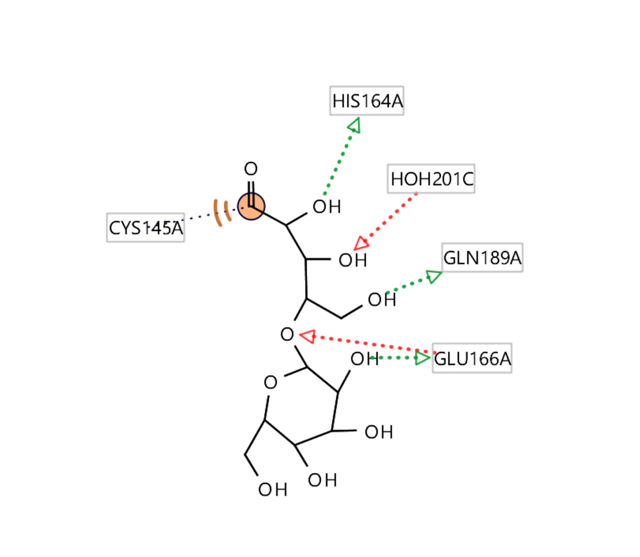

Mdl 101,146

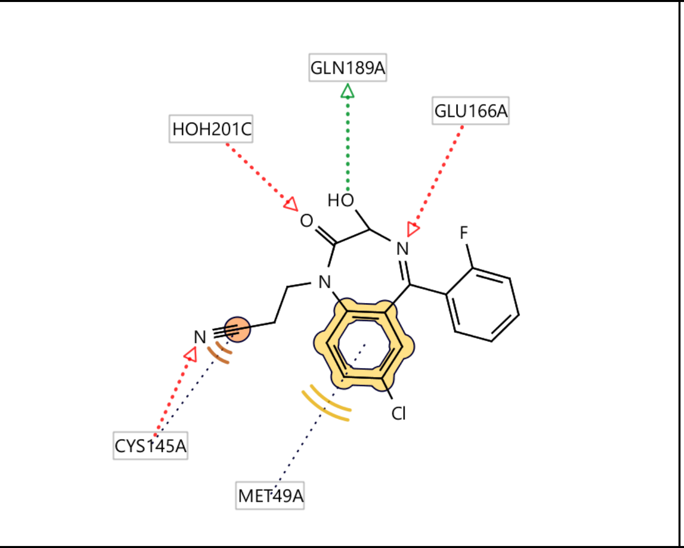

Fosamprenavir

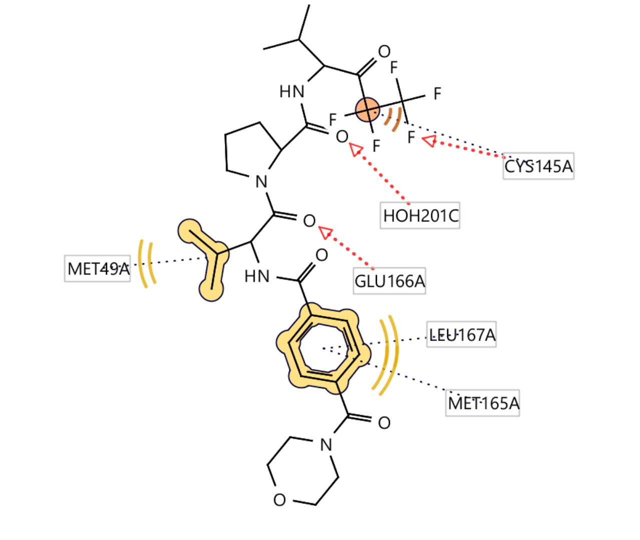

Bicalutamide

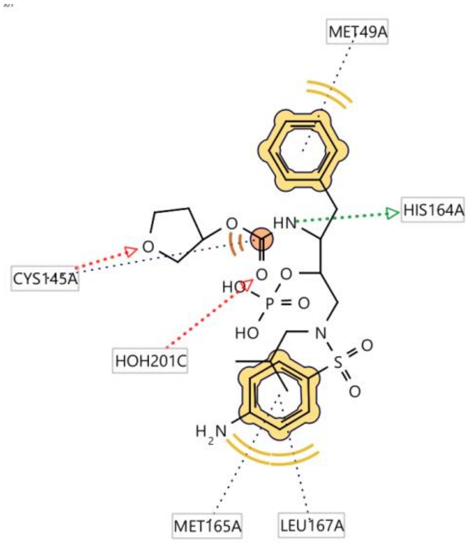	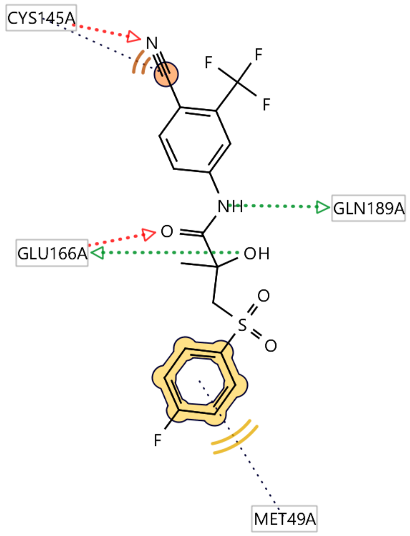
**DB04293**	**Atazanavir**
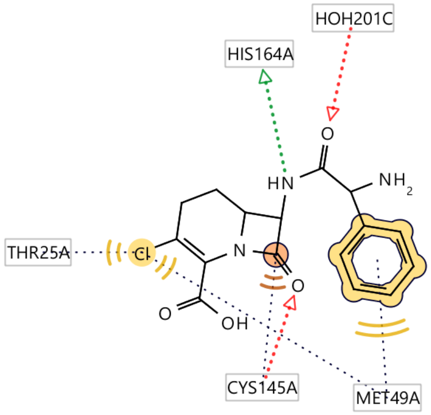	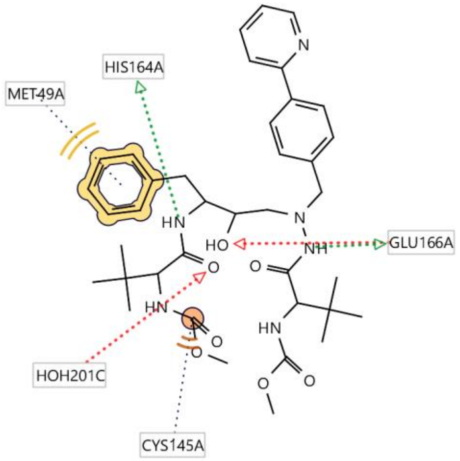
**BMS-488043**	**DB04232**
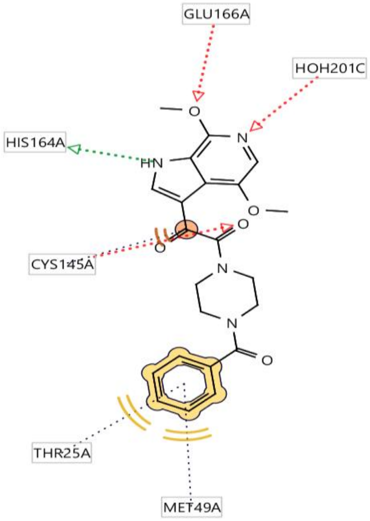	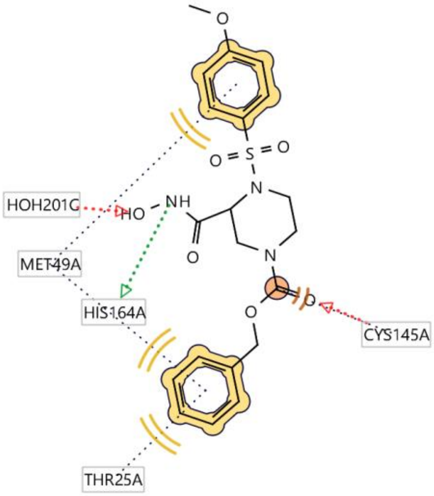
**Cephaloglycin**	**Mupirocin**

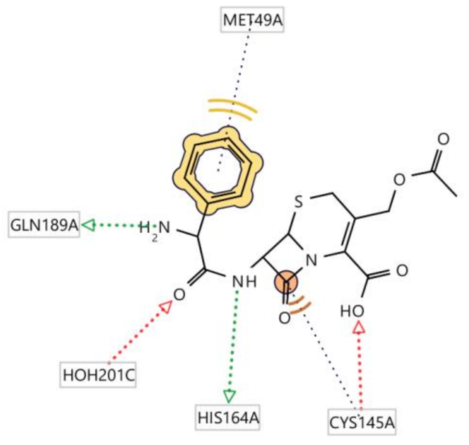	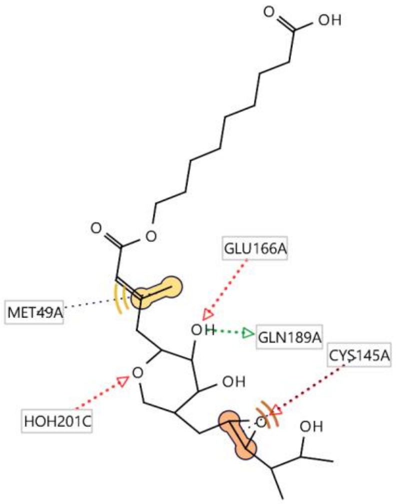
**Cefditoren**	**Cabazitaxel**
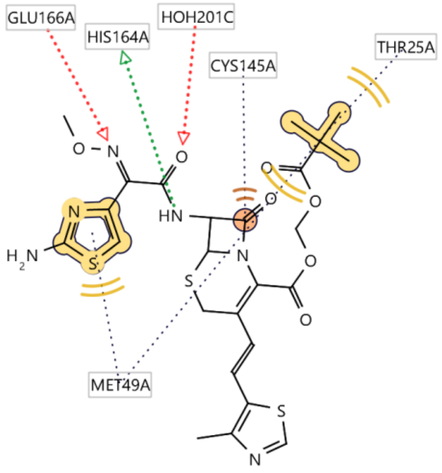	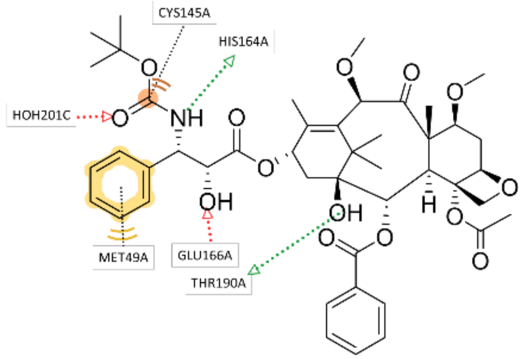

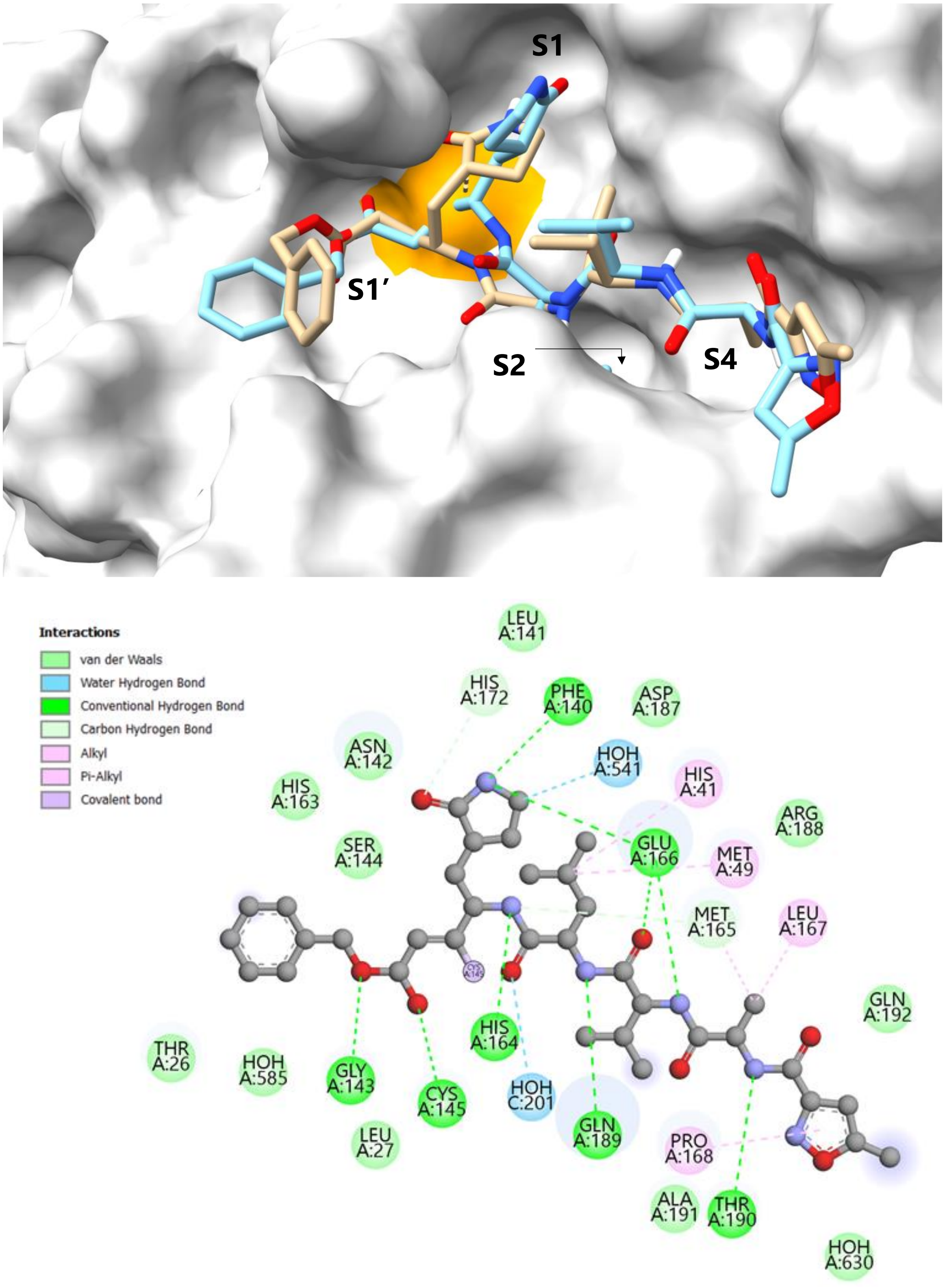

Figure S1. N3 redocking over SARS-CoV-2 M^{pro} (PDB ID: 7BQY). In blue the co-crystallized inhibiter is observed, in brown the inhibiter coupled in MOE, and in the orange color Cys145 stands out (RMSD = 0.65).

Table S2. Therapeutic applications of the best ranked drugs in virtual screening structure based.

Drug	Therapeutic indications	Target	Status	Electrophilic warhead	ΔG_{cov}
INHIBITOR N3	*SARS-CoV infection*	*SARS Mpro*	*Experimental*	*Michael-type acceptor*	-10.14
INHIBITOR N1	*SARS-CoV infection*	*3C-like proteinase*	*Experimental*	*Michael-type acceptor*	-8.51
INHIBITOR N9	*SARS-CoV infection*	*SARS Mpro*	*Experimental*	*Michael-type acceptor*	-8.22
INHIBITOR I2	*SARS-CoV infection*	*3C-like proteinase*	*Experimental*	*Michael-type acceptor*	-7.99
IXAZOMIB	*Multiple myeloma*	*20S proteasome*	*Approved*	*Boronic acid*	-5.85
CALPAIN INH I	*Acute and chronic inflammation*	*Calpain I; cathepsin B and L*	*Experimental*	*Aldehyde*	-7.40
DB08119	*Unknow*	*Chymotrypsinogen B*	*Experimental*	*Ketone*	-6.24
DB03984	*Hyperimmune response*	*Cathepsin S*	*Experimental*	*Aldehyde*	-7.37
DB07224	*Tumor metastatis; pulmonary emphysema; bone resorption*	*Cathepsin B*	*Experimental*	*Epoxide*	-6.63
DB07225	*Tumor metastatis; pulmonary emphysema; bone resorption*	*Cathepsin B*	*Experimental*	*Epoxide*	-6.59
AMPRENAVIR	*HIV infection*	*HIV-1 protease*	*Approved*	*Carbamate*	-5.82
OPROZOMIB	*Solid tumors; multiple Myeloma; Waldenstrom Macroglobulinemia*	*20S proteasome*	*Investigational*	*Epoxide*	-7.68
CARFILZOMIB	*Multiple myeloma*	*20S proteasome*	*Approved*	*Epoxide*	-8.47
DB07299	*Thrombosis*	*Coagulation factor XI*	*Experimental*	*Ketone*	-7.30
DB03767	*Autoimmune diseases*	*Cathepsin S*	*Experimental*	*Carbonitrile*	-7.21
DB04234	*Osteoporosis*	*Cathepsin K*	*Experimental*	*Aldehyde*	-7.30
BOCEPREVIR	*Chronic Hepatitis C*	*NS3/4A protease*	*Approved*	*Diketone*	-8.20
VABORBACTAM	*Urinary tract infections*	*Beta-lactamase*	*Approved*	*Oxaborole*	-5.93
DB07749	*Unknow*	*Chymotrypsinogen B*	*Experimental*	*Aldehyde*	-6.39

CEFTAROLINE	*Bacterial infections*	*Penicillin-binding proteins*	*Approved*	*β-lactam ring*	-8.56
TELAPREVIR	*Chronic Hepatitis C*	*NS3/4A protease*	*Approved*	*Diketone*	-8.34
DB07160	*Tumor metastatis; pulmonary emphysema; bone resorption*	*Cathepsin B*	*Experimental*	*Epoxide*	-6.11
PREDNISONE	*Inflammation; immune-mediated reactions*	*Glucocorticoid receptor*	*Approved*	*Michael-type acceptor*	-4.18
DARUNAVIR	*HIV infection*	*HIV-1 protease*	*Approved*	*Carbamate*	-6.75
PREXASERTIB	*Metastatic Castration-Resistant Prostate Cancer; leukemia*	*Protein kinase regulatory subunit*	*Investigational*	*Carbonitrile*	-5.25
TEMOCILLIN	*Liver dysfunction; urinary tract infection*	*Penicillin-binding proteins*	*Approved*	*β-lactam ring*	-7.13
CIMETIDINE	*Gastroesophageal reflux*	*Histamine H2 receptor*	*Approved*	*Carbonitrile*	-5.33
SCOPOLAMINE	*Motion sickness*	*Muscarinic acetylcholine receptors*	*Approved*	*Epoxide*	-5.55
FLUOXYMESTERONE	*Hypogonadism in males; breast neoplasms*	*Androgen receptor; Estrogen receptor alpha*	*Approved*	*Michael-type acceptor*	-4.18
METHSCOPOLAMINE	*Peptic ulcer*	*Muscarinic acetylcholine receptors*	*Approved*	*Epoxide*	-4.97
DB08614	*Ineffectively elastolytic activity*	*Chymotrypsin-like elastase family member 1*	*Experimental*	*Ketone*	-6.86
FLOVAGATRAN	*Thrombosis*	*Prothrombin*	*Investigational*	*Boronic acid*	-7.15
FELYPRESSIN	*Vasoconstrictor used in local anaesthetic injections*	*Vasopressin V1a receptor*	*Experimental*	*Disulfide bridge*	-8.79
DB07987	*Degenerative diseases; inflammatory conditions*	*Stromelysin-1*	*Experimental*	*Carbamate*	-5.94
AZTREONAM	*Sensitive gram-negative bacteria*	*Penicillin-binding protein 3*	*Approved*	*Monocyclic β-lactam*	-7.00
PREDNISOLONE	*Adrenocortical insufficiency; inflammatory conditions*	*Glucocorticoid receptor*	*Approved*	*Michael-type acceptor*	-5.06
RIOCIGUAT	*Pulmonary arterial hypertension*	*Guanylate cyclase soluble subunit alpha-2*	*Approved*	*Carbamate*	-5.72

TIOTROPIUM	*Chronic obstructive pulmonary disease*	*Muscarinic acetylcholine receptors*	*Approved*	*Epoxide*	-5.70
CMX-2043	*non-ST-segment elevation myocardial infarction (NSTEMI); unstable Angina; coronary disease*	*Insulin-Like Receptor; Creatine Kinase; Tyrosine Kinase Non Receptor 1*	*Investigational*	*disulfide bond*	-6.81
GAXILOSE	*Diagnostic of lactose intolerance*	*Lactase*	*Approved*	*Aldehyde*	-5.30
CINOLAZEPAM	*Sleep disorders*	*GABA(A) Receptor*	*Experimental*	*Carbonitrile*	-5.48
Mdl 101,146	*Ineffectively elastolytic activity*	*Chymotrypsin-like elastase family member 1*	*Experimental*	*α-halo ketone*	-7.49
FOSAMPRENAVIR	*Postexposure prophylaxis of HIV infection*	*HIV-1 protease*	*Approved*	*Carbamate*	-7.32
BICALUTAMIDE	*Stage D2 metastatic carcinoma of the prostate*	*Androgen receptor*	*Approved*	*Carbonitrile*	-5.46
DB04293	*Unknow*	*Beta-lactamase*	*Experimental*	*β-lactam ring*	-6.19
ATAZANAVIR	*HIV infection*	*HIV-1 protease*	*Approved*	*Carbamate*	-8.51
BMS-488043	*HIV infection*	*Free fatty acid receptor 4*	*Investigational*	*Diketone*	-6.97
DB04232	*Osteoarthritis; tumor metastasis; corneal ulceration*	*Stromelysin-1*	*Experimental*	*Carbamate*	-5.89
CEPHALOGLYCIN	*Bacterial infections*	*Penicillin-binding protein 1B*	*Approved*	*β-lactam ring*	-6.72
MUPIROCIN	*Impetigo; secondary skin infections*	*Isoleucine--tRNA ligase*	*Approved*	*Epoxide*	-7.03
CEFDITOREN	*Bacterial infections of the skin and respiratory tract*	*Penicillin-binding protein 2B and 1A*	*Approved*	*β-lactam ring*	-6.93
CABAZITAXEL	*Hormone-refractory metastatic prostate cancer*	*Tubulin alpha-4A and beta-1 chain*	*Approved*	*Carbamate*	-6.52

Bibliographic references of the drugs are reported in databases from:
https://www.drugs.com/
https://go.drugbank.com/
https://pubchem.ncbi.nlm.nih.gov/
https://www.kegg.jp/

Table S3. Drugs ordered according to the consensus score (calculation described in the methodology).

Drug	E_c	E_{BS}	S_{PF}	$-\Delta G_{ncov}$	$-\Delta G_{cov}$	CsScore
INHIBITOR N3	5815.39	5034.60	106.93	7.7	10.1	412.82
VABORBACTAM	453.04	10.58	73.71	6.5	5.9	436.08
INHIBITOR N1	3819.69	3132.36	106.9	6.8	8.5	454.83
DB04234	544.49	62.13	75.34	6.1	7.3	487.59
DB03456	584.84	103.11	75.87	6.8	6.1	496.79
CIMETIDINE	589.49	120.44	65.99	5.3	5.3	503.00
IXAZOMIB	674.98	170.89	83.9	6.7	5.9	521.07
CMX-2043	634.24	156.96	65.45	5.9	6.8	523.19
DB07224	659.17	156.99	76.48	6.1	6.6	529.37
DB07225	705.99	204.10	76.48	5.8	6.6	540.42
SCOPOLAMINE	646.78	122.54	65.98	6.3	5.6	558.11
DB04293	704.77	152.09	65.17	7.3	6.2	596.86
BOCEPREVIR	824.16	266.36	75.28	5.4	8.2	613.41
DB07299	934.01	398.77	75.65	6.5	7.3	621.90
DARUNAVIR	861.32	340.83	66.32	7.5	6.8	625.83
CALPAIN INHIBITOR-1	1127.00	630.46	77.81	5.1	7.4	626.71
INHIBITOR N9	2796.46	2216.40	96.23	6.3	8.2	649.65
BICALUTAMIDE	932.83	376.99	65.19	7.4	5.5	678.69
DB03767	1074.14	504.95	75.52	6.6	7.2	682.37
PREXASERTIB	921.30	339.96	66.27	7.5	5.3	687.56
RIOCIGUAT	955.36	348.80	65.57	8.3	5.7	717.46
DB08614	1151.02	623.04	65.93	7.3	6.9	730.91
INHIBITOR I2	1770.67	1112.63	86.48	7.0	8.0	795.50
AMPRENAVIR	1324.18	679.32	76.23	7.8	5.8	795.95
DB07160	1420.27	924.43	66.4	5.2	6.1	798.94
AZTREONAM	1482.14	944.90	65.62	6.8	7.0	853.04
TIOTROPIUM	1466.31	858.19	65.57	7.0	5.7	895.27
TEMOCILLIN	1660.75	1131.23	66.11	6.8	7.1	903.68
FLUOXYMESTERONE	1733.89	1110.31	65.98	7.2	4.2	993.80
DB08119	2619.91	2071.81	77.8	6.2	6.2	998.36
PREDNISONE	1738.53	1095.73	66.34	7.3	4.2	1004.01
FLOVAGATRAN	1892.44	1325.16	65.86	7.0	7.2	1010.37
METHSCOPOLAMINE	2070.99	1476.45	65.98	7.0	5.0	1088.93
DB03984	2930.08	2337.41	76.82	6.7	7.4	1123.67
PREDNISOLONE	2120.64	1507.06	65.59	7.8	5.1	1123.72
OPROZOMIB	2696.86	2033.90	76.12	6.3	7.7	1138.01
DB07987	2342.50	1777.82	65.72	6.0	5.9	1166.27
DB04232	2367.43	1768.70	65.1	6.9	5.9	1207.68
BMS-488043	2446.41	1800.92	65.15	7.9	7.0	1263.42

DB07749	3106.40	2577.53	67.84	6.1	6.4	1349.33
GAXILOSE	2999.85	2334.44	65.45	6.5	5.3	1464.24
CEFTAROLINE	3252.33	2556.58	66.62	7.1	8.6	1538.70
CINOLAZEPAM	3451.69	2514.84	65.40	7.4	5.5	1798.56
CEPHALOGLYCIN	4091.77	3432.94	64.48	7.6	6.7	1868.98
CEFDITOREN PIVOXIL	4352.81	3465.77	63.78	6.5	6.9	2133.78
Mdl 101,146	4813.17	4065.30	65.33	7.2	7.5	2147.71
TELAPREVIR	5196.13	4479.26	66.43	6.2	8.3	2210.90
CARFILZOMIB	6849.93	5948.19	76.03	8.2	8.5	2314.85
MUPIROCIN	5761.68	4899.57	63.87	6.7	7.0	2623.56
FOSAMPRENAVIR	7505.59	6894.18	65.33	7.2	7.3	2992.14
ATAZANAVIR	8900.26	7923.60	65.17	5.4	8.5	3727.38
CABAZITAXEL	9132.50	7750.80	63.67	6.7	6.5	4189.15
FELYPRESSIN	10838.66	9699.06	65.83	6.8	8.8	4443.51

Table S4. 2D structure of the best ranked drugs according to the consensus score.

N3
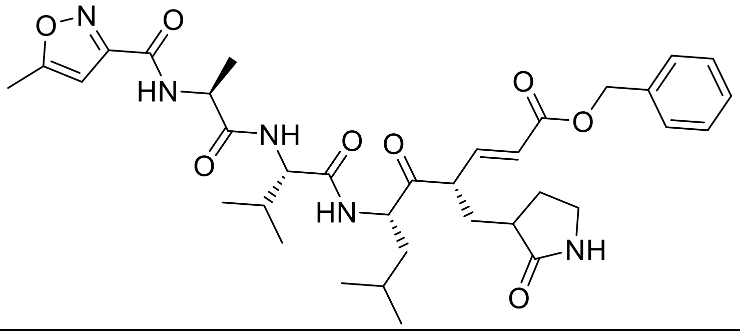
**VABORBACTAM**
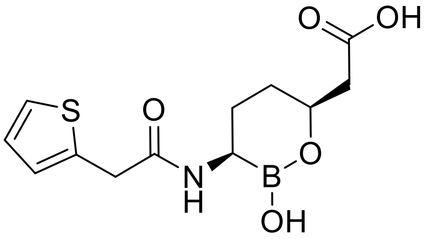
**N1**
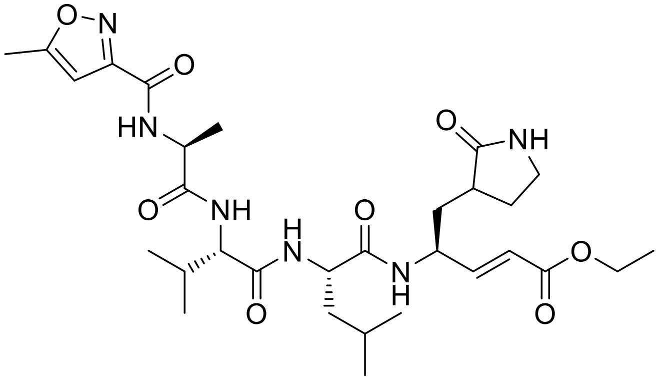

**DB04234**
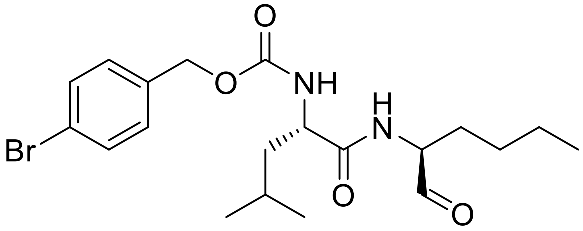
**DB03456**
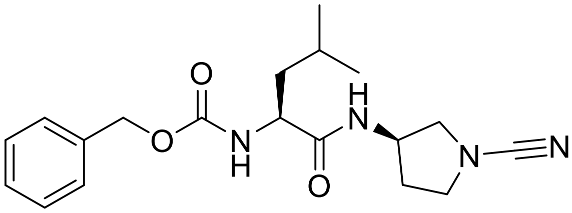
**CIMETIDINE**
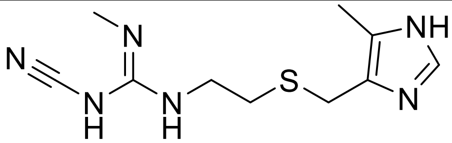
**IXAZOMIB**
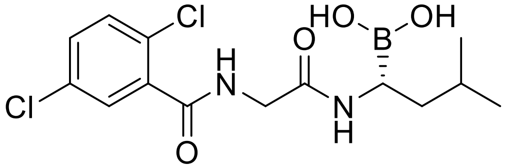
**CMX-2043**
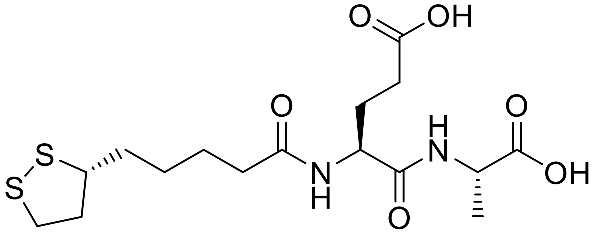
**DB07224**
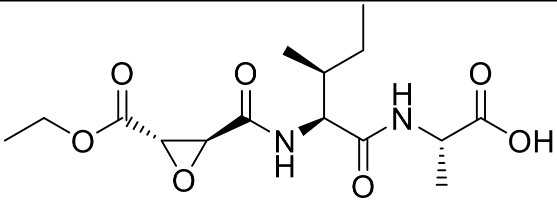
**DB07225**
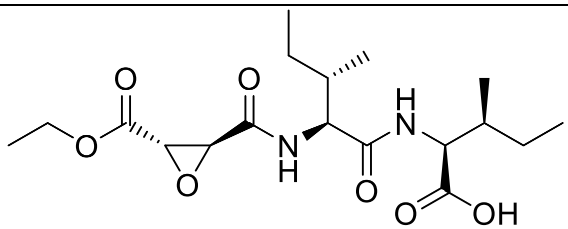

**SCOPOLAMINE**
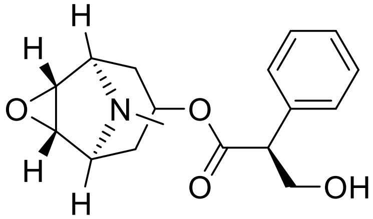
**DB04293**
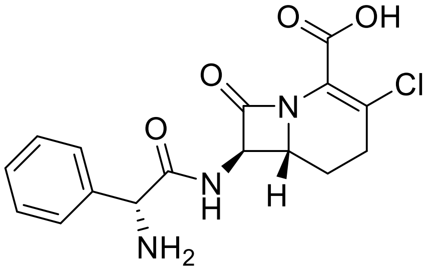
**BOCEPREVIR**
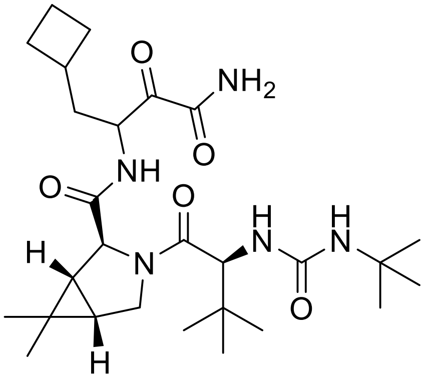
**DB07299**
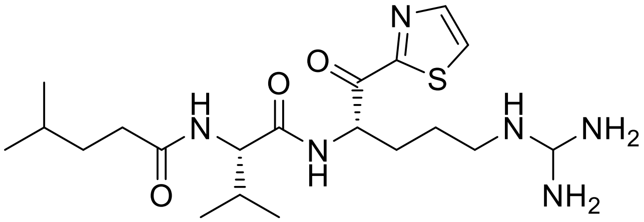
**DARUNAVIR**
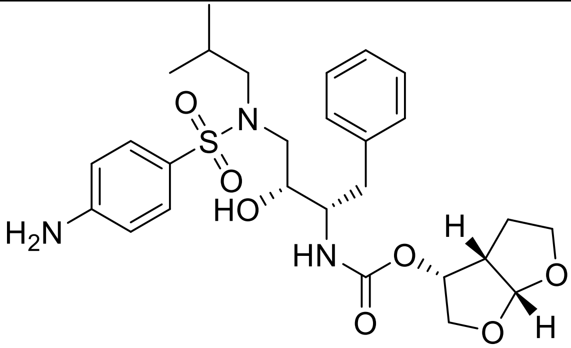
**CALPAIN INHIBITOR-1**

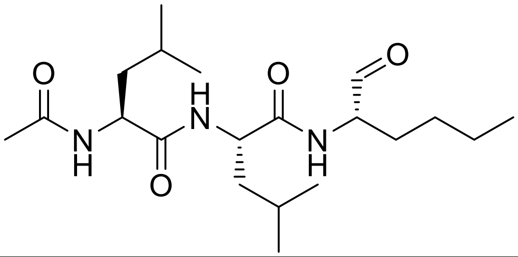
**N9**
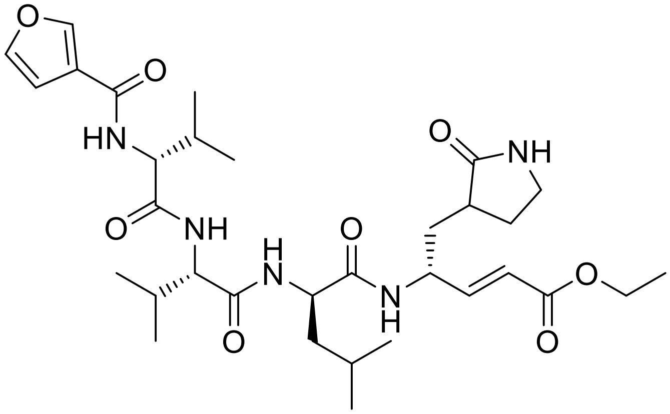
**BICALUTAMIDE**
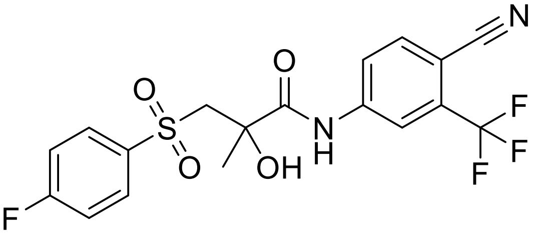
**DB03767**
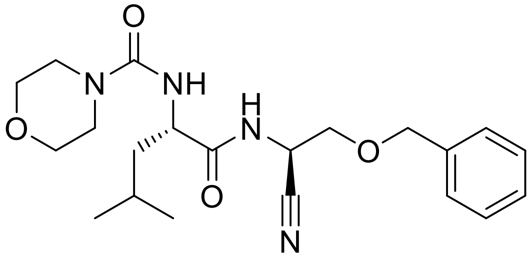
**PREXASERTIB**
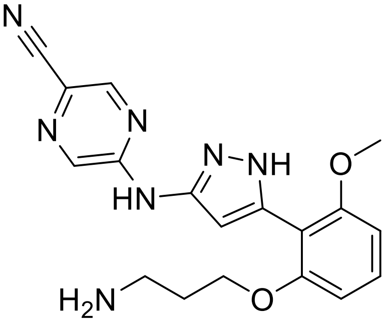
**RIOCIGUAT**

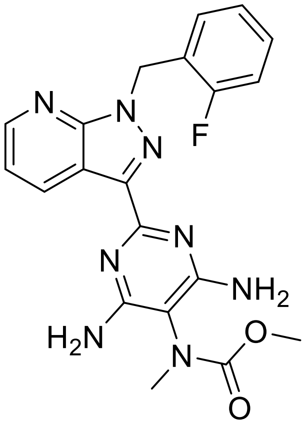

DB08614

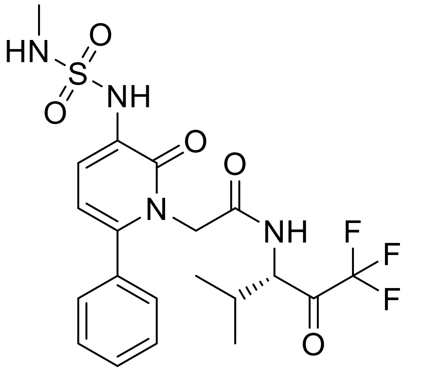

I2

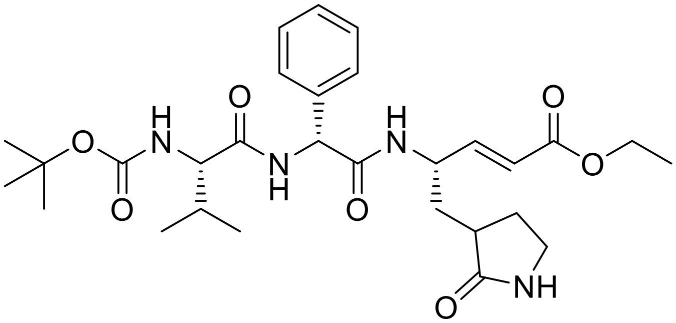

AMPRENAVIR

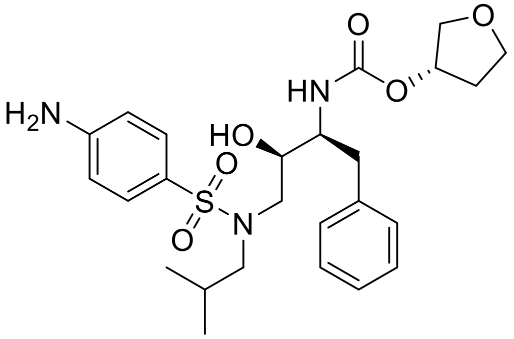

DB07160

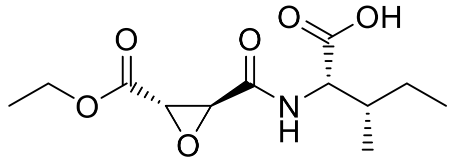

AZTREONAM

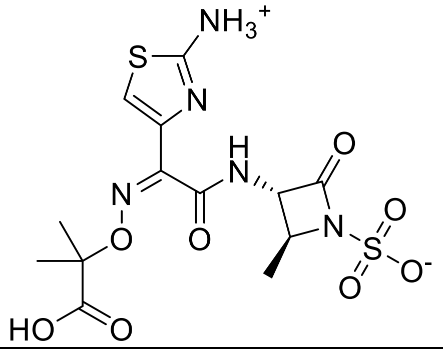

TIOTROPIUM

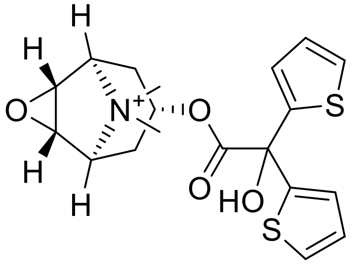

TEMOCILLIN

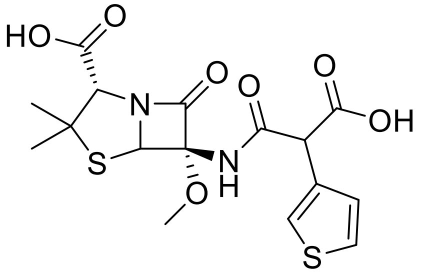

FLUOXYMESTERONE

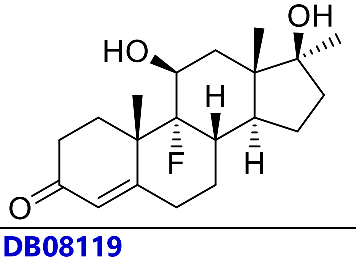

DB08119

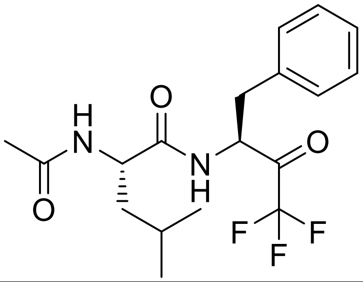
**PREDNISONE**
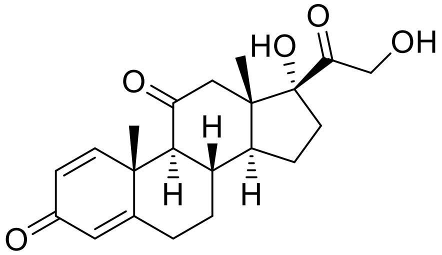
**FLOVAGATRAN**
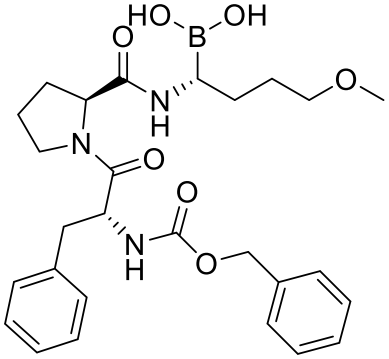
**METHSCOPOLAMINE**
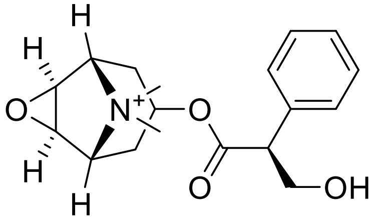
**DB03984**
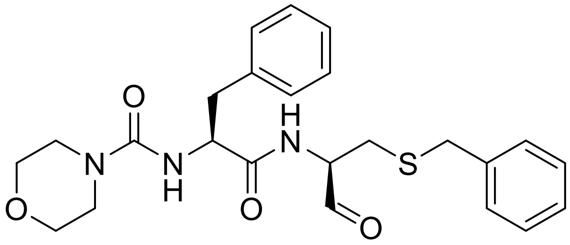
**PREDNISOLONE**

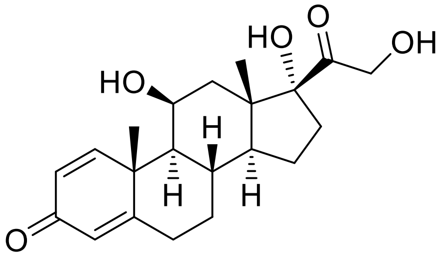

OPROZOMIB

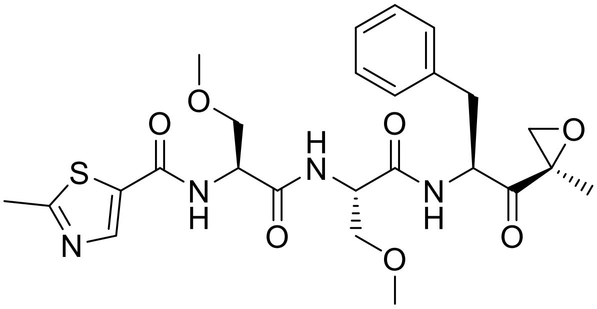

DB07987

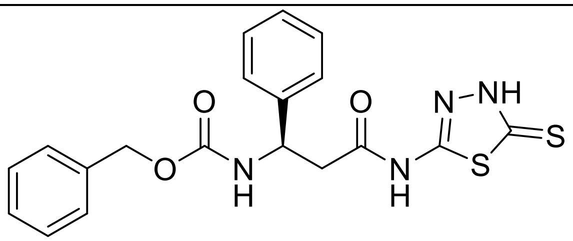

DB04232

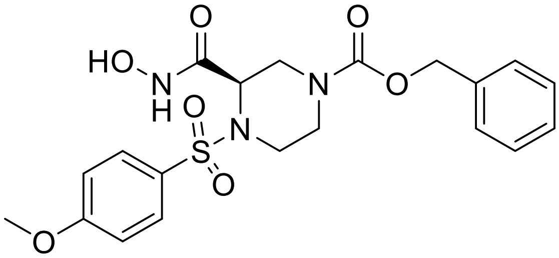

BMS-488043

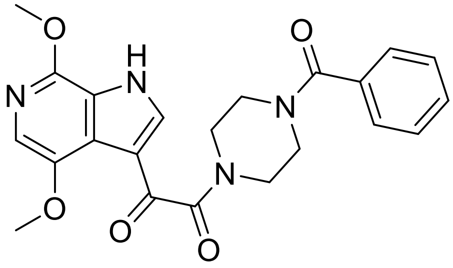

DB07749

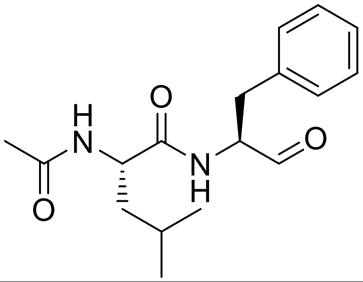

GAXILOSE

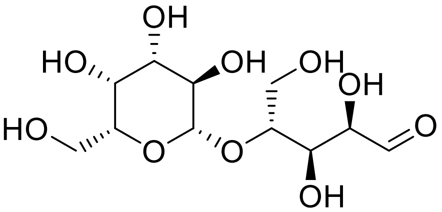

CEFTAROLINE

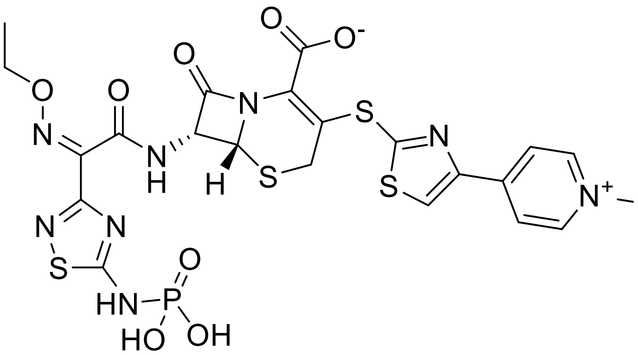

CINOLAZEPAM

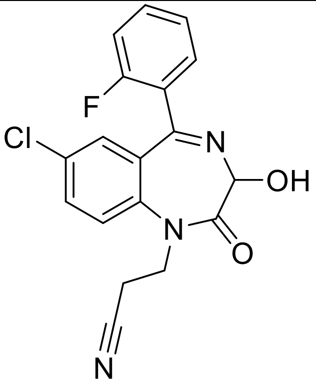

CEPHALOGLYCIN

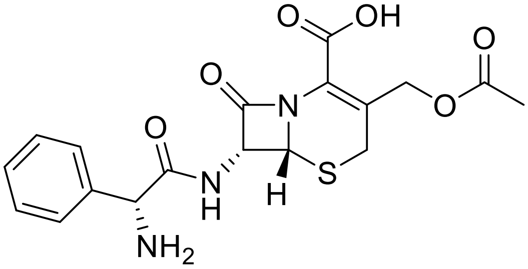

CEFDITOREN PIVOXIL

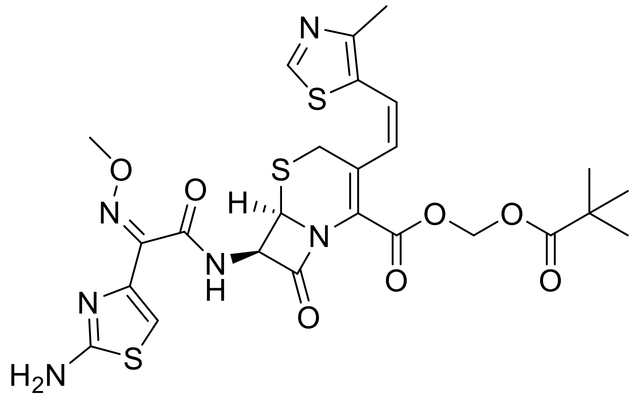

Mdl 101,146

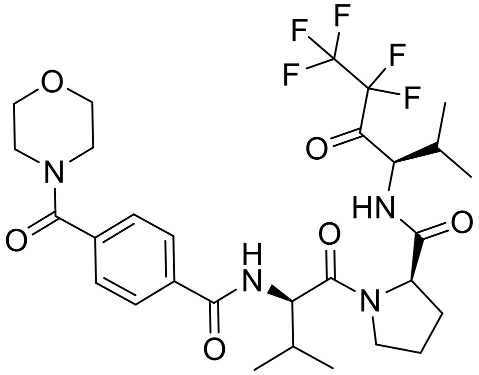

TELAPREVIR

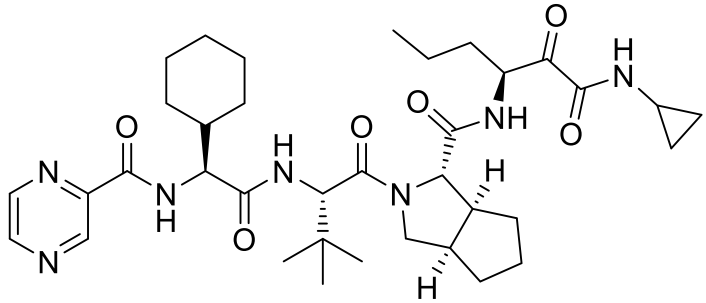

CARFILZOMIB

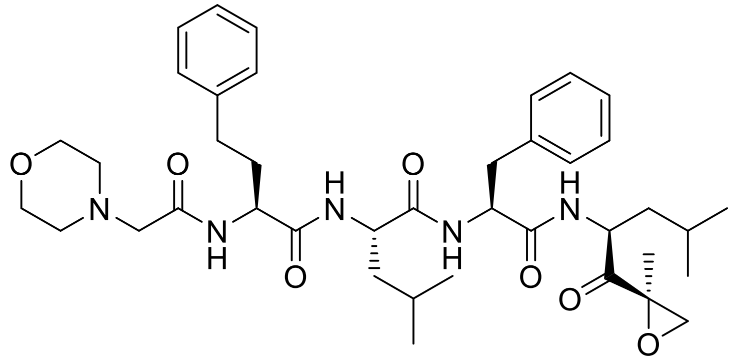

MUPIROCIN

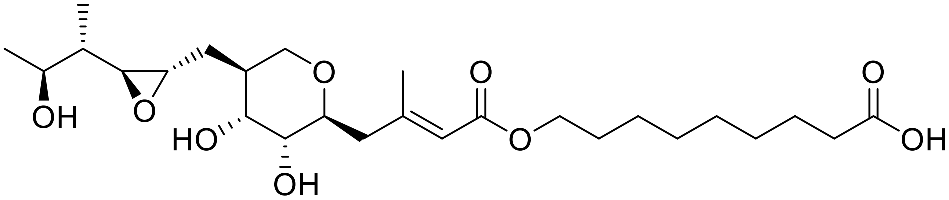

FOSAMPRENAVIR

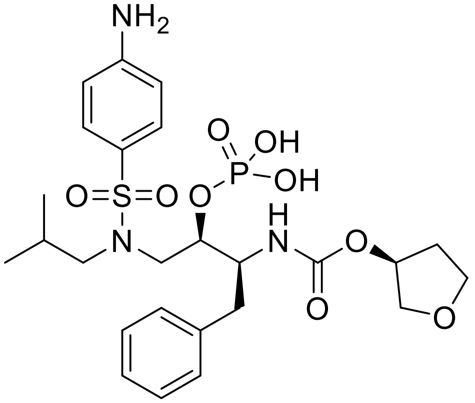

ATAZANAVIR

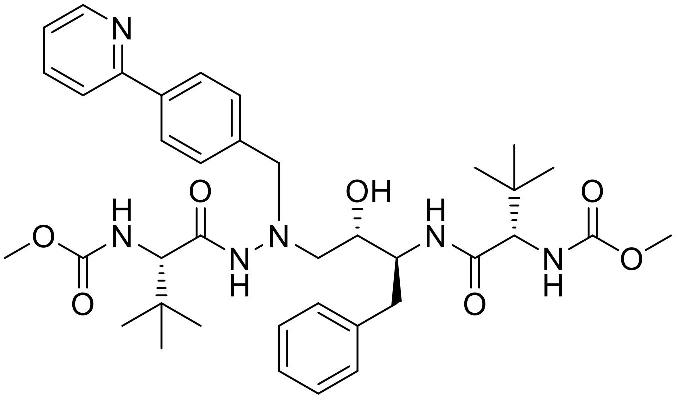

CABAZITAXEL

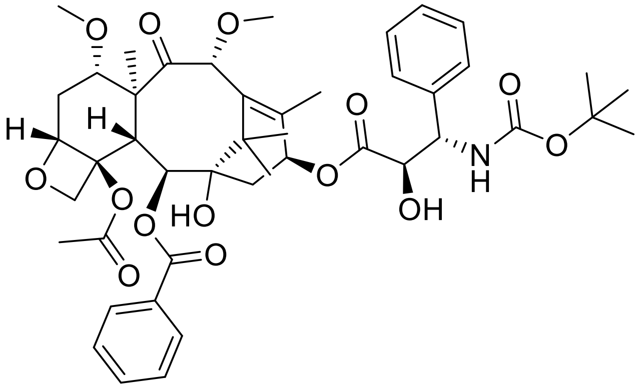

FELYPRESSIN

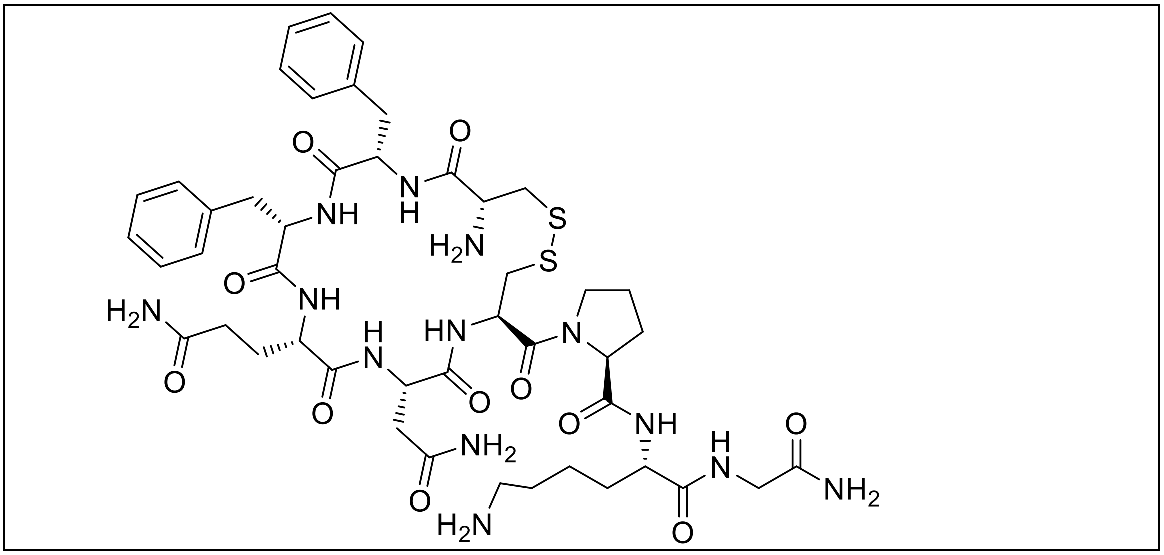

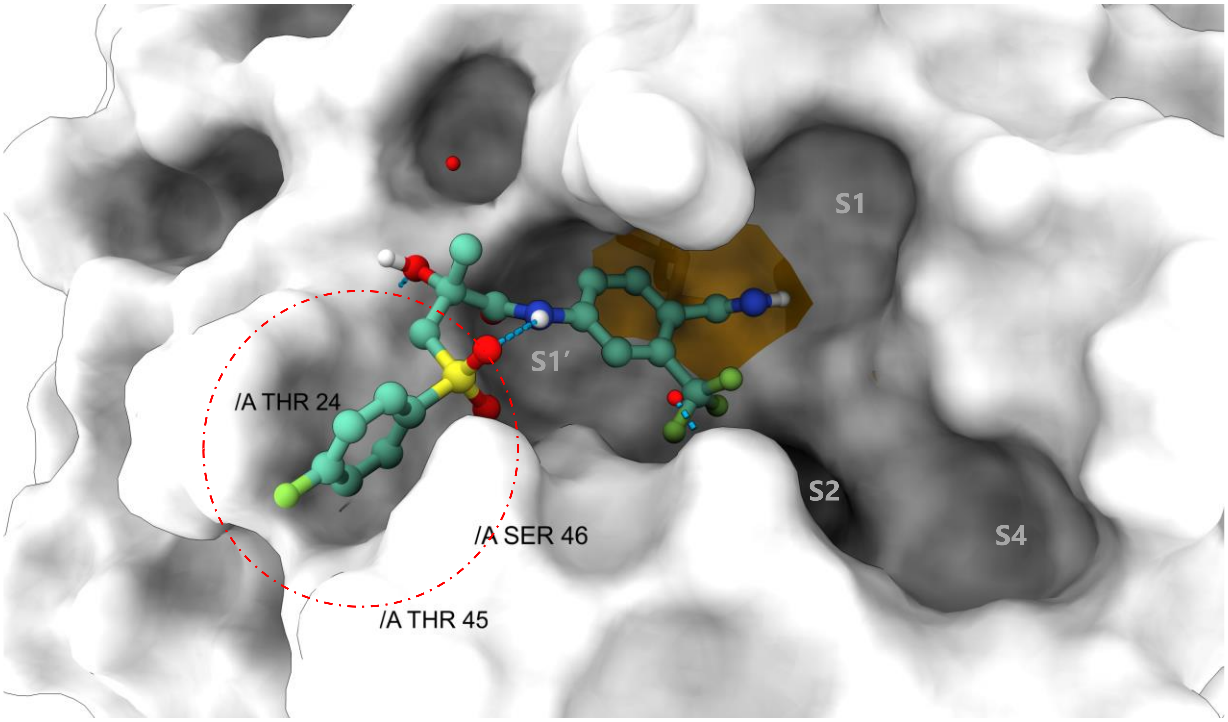

Figure S2. Binding mode of Bicatulamide in SARS-CoV-2 M^{pro}, where the subsite occupied by the 4-fluorobenzenesulfonyl substituent is highlighted.
